# Membrane Recognition and Dynamics of the RNA Degradosome

**DOI:** 10.1371/journal.pgen.1004961

**Published:** 2015-02-03

**Authors:** Henrik Strahl, Catherine Turlan, Syma Khalid, Peter J. Bond, Jean-Marie Kebalo, Pascale Peyron, Leonora Poljak, Marie Bouvier, Leendert Hamoen, Ben F. Luisi, Agamemnon J. Carpousis

**Affiliations:** 1 Centre for Bacterial Cell Biology, Institute for Cell and Molecular Biosciences, Newcastle University, Newcastle, United Kingdom; 2 Laboratoire de Microbiologie et Génétique Moléculaires, UMR 5100, CNRS et Université Toulouse III, Toulouse, France; 3 School of Chemistry, University of Southampton, Southampton, United Kingdom; 4 Bioinformatics Institute (A*STAR), Singapore; 5 Department of Biological Sciences, National University of Singapore, Singapore; 6 Swammerdam Institute for Life Sciences (SILS), University of Amsterdam, Amsterdam, the Netherlands; 7 Department of Biochemistry, University of Cambridge, Cambridge, United Kingdom; University of Geneva Medical School, SWITZERLAND

## Abstract

RNase E, which is the central component of the multienzyme RNA degradosome, serves as a scaffold for interaction with other enzymes involved in mRNA degradation including the DEAD-box RNA helicase RhlB. Epifluorescence microscopy under live cell conditions shows that RNase E and RhlB are membrane associated, but neither protein forms cytoskeletal-like structures as reported earlier by Taghbalout and Rothfield. We show that association of RhlB with the membrane depends on a direct protein interaction with RNase E, which is anchored to the inner cytoplasmic membrane through an MTS (Membrane Targeting Sequence). Molecular dynamics simulations show that the MTS interacts with the phospholipid bilayer by forming a stabilized amphipathic α-helix with the helical axis oriented parallel to the plane of the bilayer and hydrophobic side chains buried deep in the acyl core of the membrane. Based on the molecular dynamics simulations, we propose that the MTS freely diffuses in the membrane by a novel mechanism in which a large number of weak contacts are rapidly broken and reformed. TIRFm (Total Internal Reflection microscopy) shows that RNase E in live cells rapidly diffuses over the entire inner membrane forming short-lived foci. Diffusion could be part of a scanning mechanism facilitating substrate recognition and cooperativity. Remarkably, RNase E foci disappear and the rate of RNase E diffusion increases with rifampicin treatment. Control experiments show that the effect of rifampicin is specific to RNase E and that the effect is not a secondary consequence of the shut off of *E. coli* transcription. We therefore interpret the effect of rifampicin as being due to the depletion of RNA substrates for degradation. We propose a model in which formation of foci and constraints on diffusion arise from the transient clustering of RNase E into cooperative degradation bodies.

## Author Summary

Recent discoveries that two ribonucleases with major roles in mRNA degradation, RNase E of *Escherichia coli* and RNase Y of *Bacillus subtilis*, are localized to the inner cytoplasmic membrane suggest that spatial separation of transcription and mRNA degradation are general features of the bacterial cell. Here we show that RNase E rapidly diffuses over the entire inner membrane forming short-lived foci. Results of molecular dynamics simulations lead us to suggest that RNase E interacts with the lipid membrane by a novel mechanism permitting a high degree of translational freedom. We show that RNA substrate is necessary for the formation of RNase E foci and that formation of foci correlates with constraints on the diffusion of RNase E. We therefore propose that foci are degradation bodies in which several RNase E molecules engage an RNA substrate. The sequestration of the mRNA degradation machinery to the inner cytoplasmic membrane has important consequences for mRNA turnover. This organization likely favors formation of polyribosomes on nascent transcripts before they are exposed to the degradation machinery. Rapid diffusion of RNase E on the inner cytoplasmic membrane could be part of a scanning mechanism that facilitates recognition of cytoplasmic polyribosomes and cooperative degradation of mRNA.

## Introduction

In *Escherichia coli*, *Salmonella*, and many other bacteria, RNase E makes critical contributions to general and regulated mRNA degradation [1, 2]. General mRNA degradation is the default turnover pathway, whereas regulated mRNA degradation is controlled by factors such as sRNA (small RNA) and the RNA binding protein Hfq [3, 4]. RNase E contains a large noncatalytic region that is the scaffold for the assembly of a multienzyme complex known as the RNA degradosome [5]. Recently, RNase E was shown to be localized to the inner cytoplasmic membrane by tagging with fluorescent protein [6, 7], a finding that has been corroborated for the native enzyme as well as other RNA degradosome components by proteomic analyses of the inner membrane [8, 9]. The association of RNase E with the membrane benefits organism fitness as indicated by the slow growth of strains bearing deletions or point mutations that disrupt membrane binding [6], so the interaction is likely to be functionally important. It has been postulated that membrane association physically separates sites of transcription from sites of mRNA degradation and thereby confers a time delay before the onset of decay of a transcript [10]. The general importance of the localization of RNase E has been underscored by the recent finding that RNase Y, a key ribonuclease of mRNA degradation in *Bacillus subtilis*, is also membrane-localized [11]. What makes this parallel especially striking is that RNase E and RNase Y share no common evolutionary ancestor and their functional analogy therefore arose through convergent evolution.

The basis for the interaction of RNase E with a phospholipid bilayer was established by the identification of a 15-residue MTS in the noncatalytic region that is necessary and sufficient for membrane localization [6]. The MTS, with the propensity to form an amphipathic α-helix, bears signature features conserved in RNase E homologs throughout the γ-Proteobacteria including the clustering of bulky aromatic residues on one face of the helix, small hydrophilic residues on the opposite face and the presence of basic residues flanking the hydrophobic core. Mutation of signature residues of the MTS of *E. coli* RNase E confirmed their importance for membrane localization *in vivo* and for interaction with protein-free phospholipid vesicles *in vitro* [6]. To elucidate the structural basis for RNase E recognition of the cytoplasmic membrane, we have undertaken molecular dynamics simulations with a realistic model of the *E. coli* inner membrane and the MTS peptide, and we have performed binding studies using a fluorescein-labelled derivative of this peptide. These analyses shed light on the geometry, energetics and dynamics of the interaction of the MTS with the lipid bilayer.

Recent reports suggest that RNase E and RhlB, which is a DEAD-box RNA helicase component of the RNA degradosome, form a membrane-associated cytoskeletal-like structure, and that RhlB localizes to the cytoskeleton independently of RNase E [7, 12, 13]. The question therefore arises how RNase E and RhlB interact *in vivo*, and to what degree RNase E and RhlB are free to diffuse on the inner cytoplasmic membrane. To address this question, we have conducted microscopy studies in live cells in which RNase E and RhlB were tagged with fluorescent protein. Under live cell conditions, the association of RhlB with the membrane depends on its interaction with RNase E. In addition, we observe that RNase E rapidly diffuses on the inner cytoplasmic membrane forming transient foci. The likely impact of the membrane dynamics of RNase E on its access to RNA substrates and the coordinated activities of the degradosome will be discussed.

## Results

### Recruitment of RhlB to the inner cytoplasmic membrane

Although there is evidence that the DEAD-box RNA helicase RhlB associates with RNase E through a direct protein-to-protein interaction [5, 14, 15], recent reports have suggested that RhlB by itself can form a membrane-associated cytoskeletal-like structure [12, 13]. We therefore explored the structural requirement for the localization of RhlB to the inner cytoplasmic membrane by constructing strains in which RNase E and RhlB were tagged at their C-terminal ends with mCherry and CFP (Cyan Fluorescence Protein), respectively. These constructs are functional single copy chromosome replacements in the NCM3416 background, which is a wild type *E. coli* K12 strain [16]. Additional constructs contain variants of RNase E-mCherry in which the MTS, protein Scaffold (Sca) or HBS (Helicase Binding Site) were deleted based on previous work mapping these sites [17].

**Figure 1:**
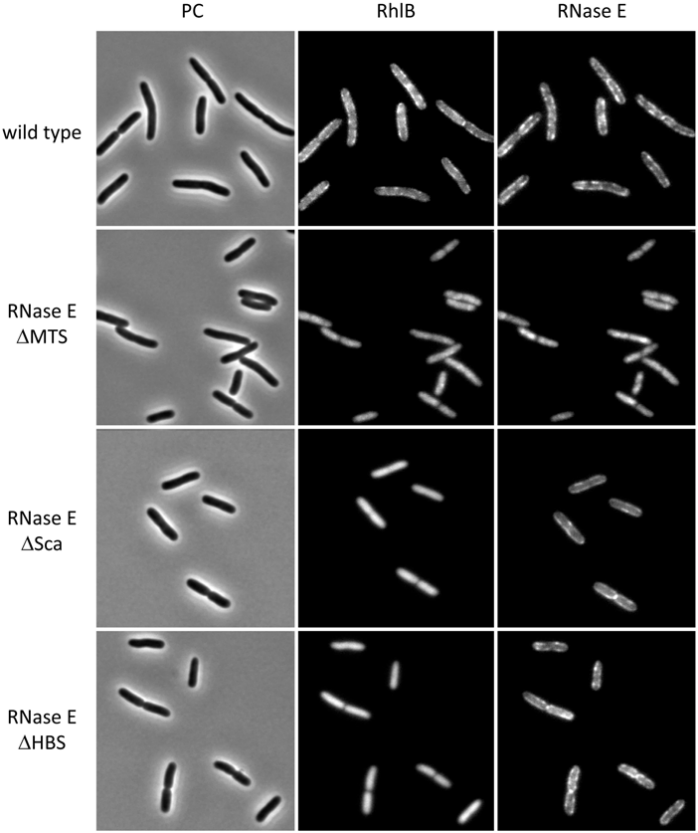
Membrane localization of RhlB depends on a direct protein-to-protein interaction with RNase E. Gallery of micrographs showing images of cell expressing RNase E-mCherry and RhlB-CFP. Gene fusions are present as functional single copy chromosome replacements in the NCM3416 background. PC = phase contrast. In this figure, a few cells were chosen from a larger field (S1 Fig.). Wild type, Kti200 strain encoding RNase E-mCherry and RhlB-CFP. RNase E ΔMTS, Kti515 strain encoding a variant with deletion of the segment corresponding to the MTS (RNase E Δ567–582) and RhlB-CFP. RNase E ΔSca, Kti740 strain encoding a variant with deletion of the protein scaffold (RNase E Δ702–1061) and RhlB-CFP. RNase E ΔHBS, Kti738 strain encoding a variant with deletion of the HBS (RNase E Δ705–737) abd RhlB-CFP. Cultures were grown to mid logarithmic phase in MOPS-glycerol-amino acids media at 30°C. The RhlB and RNase E images were made with a 4 s exposure time.

Fig. 1 presents a gallery of micrographs showing images of strains expressing RhlB-CFP and RNase E-mCherry. In Fig. 1, a few cells were chosen from a large field (S1 Fig.). Cultures were grown to mid logarithmic phase in MOPS-glycerol-amino acids media at 30°C. Similar results were obtained in LB media and at 37°C. In the wild type strain (top panel), RNase E and RhlB are enriched in foci at the periphery of the cell. RNase E and RhlB do not co-localize in these images, which were made with a 4 s exposure time due to the weak RhlB-CFP fluorescence signal. The apparent lack of co-localization is likely due to rapid movement of RNase E under the live cell conditions used in these experiments (S2 Fig. and results below). In the second panel, RNase E ∆MTS is a variant in which the MTS has been deleted. Both RNase E and RhlB are delocalized from the periphery and the signal is cytoplasmic and diffuse. These results demonstrate that the membrane localization of RhlB depends on the MTS of RNase E. In the third panel, RNase E ∆Sca is a variant with a deletion of the scaffold, which interacts with RhlB, enolase and PNPase. Like the wild type protein, RNase E ∆Sca is localized in foci at the periphery of the cell, whereas RhlB is cytoplasmic and diffuse. These results demonstrate that the membrane localization of RhlB requires an interaction with the scaffold region of RNase E, either directly or indirectly via an interaction with another component of the RNA degradosome. In the fourth panel, RNase E ∆HBS is a variant of RNase E in which the binding site for RhlB has been deleted. RhlB is localized to the cell interior as in the RNase E ∆Sca construct. These results demonstrate that the localization of RhlB to the membrane depends on a direct protein-to-protein interaction with RNase E. Due to the limit of resolution of light microscopy, we cannot exclude the possibility that some molecules of RhlB-CFP remain membrane associated in the RNase E ∆Sca and ∆HBS constructs. We nevertheless conclude that most if not all of the membrane localization of RhlB-CFP depends on a protein interaction with RNase E and that RhlB by itself is not a membrane binding protein. Throughout the construction and imaging of these strains, as well as imaging in other strain backgrounds [6], we have not observed cytoskeletal-like structures that were reported in other studies [7, 12, 13].

### Molecular dynamics simulations of the interaction of the MTS with a phospholipid bilayer

**Figure 2:**
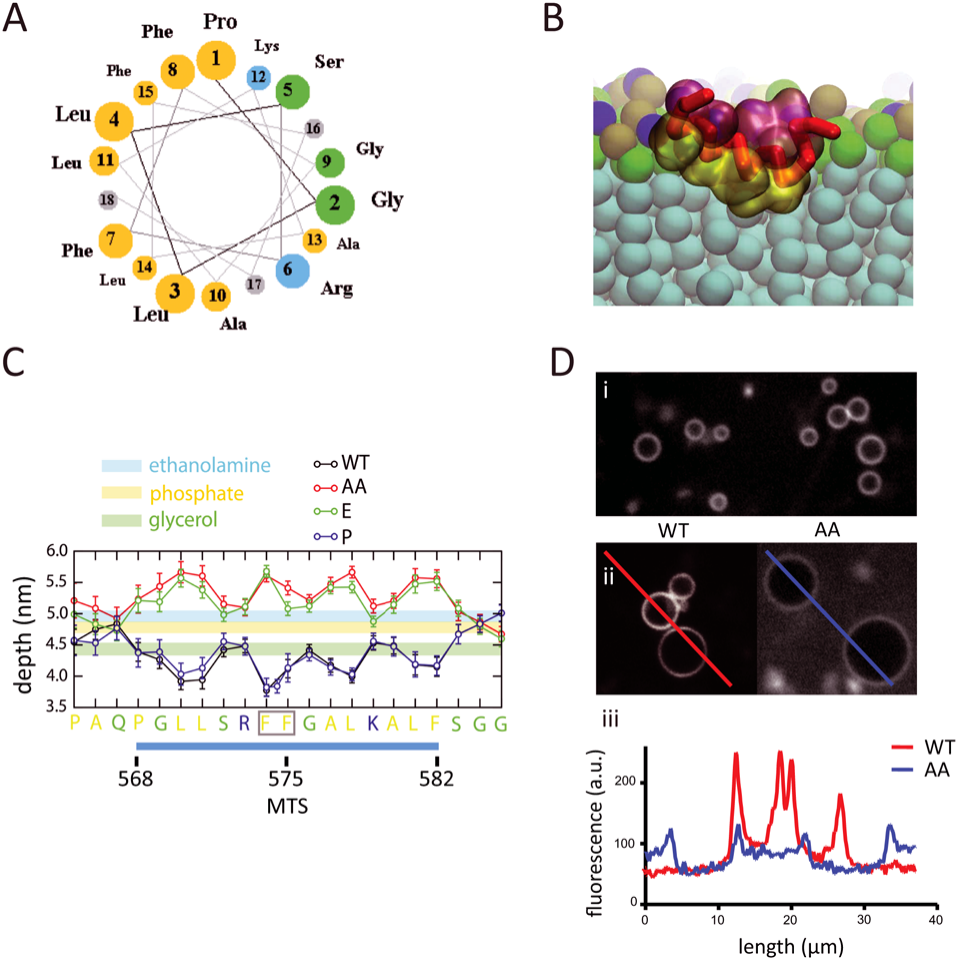
Molecular dynamics simulation of MTS interaction with phospholipid bilayer. A. Helical wheel representation of *E. coli* RNase E MTS (Membrane Targeting Sequence). The 15-residue sequence corresponds to residues 568–582 of RNase E. Yellow, hydrophobic residues; blue, basic residues; green, small hydrophilic residues. Molecular dynamics simulations were made with a slightly large peptide that corresponds to residues 565–585 of RNase E. B. Snapshot from molecular dynamics simulation of the interaction of the MTS with phospholipid bilayer. The backbone of the MTS is shown in red, hydrophobic residues in yellow and charged/hydrophilic residues in pink. Water molecules have been masked. The phospholipid bilayer has been sliced transversally (aqua, acyl interior; green, glycerol moieties; brown, phosphate moieties; turquoise, ethanolamine moieties). A molecular graphics movie of the simulation is provided (S1 Video). C. Depth of insertion of the wild type MTS peptide and variants into the phospholipid bilayer. Depth (ordinate) corresponds to distance from the plane of the phospholipid bilayer. The sequence (abscissa) corresponds to the region of RNase E containing the MTS. The sequence underlined in blue corresponds to the phylogenetically conserved element (residues 568–582 of RNase E) that was shown experimentally to have the propensity to form an α-helix upon interaction with the membrane [6]. WT = wild type sequence (black line). Sequence variants: AA, F574A/F575A (red line); E, F575E (green line); P, insertion of proline between F574 and F575 (blue line). The depth of the inserted proline is shown by the additional point on the blue line. D. Binding of fluorescein-labelled peptides corresponding to wild type MTS and AA variant (F574A/F575A) to liposomes prepared from *E. coli* lipid extracts. i) Large field image of phospholipid vesicles in the absence of peptide. ii) Vesicles in the presence of peptide. iii) Quantification of fluorescence intensity along the lines traced in ii). The higher background in the right image (AA variant) is due to unbound peptide.

To better understand the molecular basis for the interaction of the MTS with the phospholipid bilayer, and to determine what constraints, if any, this type of membrane association has on the diffusion of RNase E, we performed coarse-grain molecular dynamics simulations. We used a membrane model with realistic *E. coli* lipid composition, namely cardiolipin (CARD), dipalmitoylphosphatidylglycerol (PG) and dipalmitoylphosphatidylethanolamine (PE) in a ratio of approximately 1:2:6, respectively [18, 19]. We used a 21-residue sequence corresponding to the residues 565–585 of RNase E, which includes the 15-residue core of the MTS (Fig. 2A). This peptide was used in previous biophysical measurements of the interaction of the MTS with a phospholipid bilayer made with purified *E. coli* lipids [6]. In the coarse-grain simulation, the peptide starts in bulk solution in a helical conformation, and with time randomly encounters the phospholipid bilayer, whereupon it adheres to the membrane surface and then penetrates into the acyl interior (Fig. 2B). A molecular graphics movie of the simulation is provided (S1 Video). The simulation shows that the MTS inserts in the membrane with the helical axis oriented parallel to the bilayer plane and hydrophobic side chains buried deep into the acyl core of the lipids. The peptide interacts with approximately 50 phospholipids (S3 Fig.), in reasonable agreement with previous calorimetry measurements in which the binding isotherm was fitted to an interaction with approximately 40 phospholipids [6]. The helical conformation of the MTS was retained throughout the coarse-grain simulation. Atomistic simulations of the MTS without any restraints on the secondary structure revealed that the MTS is a stable helix in the environment of the phospholipid bilayer. This result is consistent with previous circular dichroism measurements showing that the peptide has greater propensity to form an α-helical conformation upon interaction with a phospholipid bilayer composed of *E. coli* lipids [6].

Molecular dynamics simulations were also undertaken with MTS variants that were previously studied experimentally [6]. These are a double substitution of phenylalanine with alanine (F574A/F575A); a substitution of phenylalanine with glutamic acid (F575E); and a proline insertion between residues F574 and F575. In the wild type peptide, the hydrophobic amino acid side chains penetrate into the acyl interior of the lipid bilayer, the small hydrophilic and basic amino acid side chains interact at the level of the glycerol moieties (Fig. 2C). The 3-residue N- and C-terminal extensions flanking the MTS interact at the level of the phosphate and ethanolamine moieties. Estimates of the energy of interaction (S4 Fig.) correlate with the depth of insertion of the hydrophobic amino acids into the membrane. In the F574A/F575A and F575E variants, the hydrophobic side chains protrude into the aqueous layer and the small hydrophilic and basic residues interact at the level of the ethanolamine moieties (Fig. 2C). In previous calorimetry experiments employing the corresponding peptides, no heat of interaction with lipid vesicles was detected with peptides corresponding to the F574A/F575A and F575E variants [6] suggesting that interaction of the hydrophobic side chains with the acyl core of the lipid bilayer drives the binding reaction. Microscopy of fluorescein-labelled peptides that correspond to the sequences in the molecular dynamics simulations confirms binding *in vitro* to vesicles made with purified *E. coli* lipids (Fig. 2D). The degree of fluorescence at the membrane diminishes with the F574A/F575A variant, in accord with the findings from the molecular dynamics simulations and the diminished membrane association of the corresponding RNase E variant *in vivo* [6]. The simulation with the proline insertion variant shows that it has a more favorable energy of interaction than wild-type (S4 Fig.), probably due to additional contacts with the inserted proline, which is buried deep in the acyl interior of the lipid bilayer (Fig. 2C). The results of the molecular dynamics calculations are in agreement with experimental work showing that full-length RNase E with a proline insertion at this position interacts with phospholipid vesicles *in vitro* and localizes to the cytoplasmic membrane *in vivo* [6]. The stability of the helix in the membrane and the estimated free energy explain why the proline insertion does not disrupt interaction with the phospholipid bilayer. The congruence between the previous biophysical measurements and the properties predicted by the molecular dynamics simulation validate the coarse-grain approach used here.

Since membrane composition and curvature can affect protein binding [20, 21], we asked whether these parameters are predicted to affect the interaction of the MTS with the phospholipid bilayer. The molecular dynamics simulations indicate that the MTS exhibits preferential interaction with anionic lipids (CARD and PG) in comparison to Zwitterionic lipids (PE) (S5 Fig.). This result suggests that the basic residues flanking the hydrophobic core of the amphipathic α-helix, which is a conserved feature of the MTS [6], form favorable electrostatic contacts with anionic lipids that help to stabilize the α-helix and/or increase the energy of interaction. Membrane composition could therefore affect the interaction of the MTS with the phospholipid bilayer. The molecular dynamics simulations presented here are based on planar lipid bilayers, but simulations of small lipid vesicles with curved surfaces composed of phosphatidlycholine (PC) or a realistic mixture of *E. coli* lipids gave similar results suggesting that the interaction of the MTS with the phospholipid bilayer is not sensitive to membrane curvature.

Diffusion can be important for the function of a membrane protein since it affects interactions with substrate and other proteins. In the coarse grain simulation using a 10 µs period, the rate of diffusion for the MTS on the membrane was predicted to be in the order of 10^3^ µm^2^/s. We know of no other study of a bacterial peripheral membrane protein that can be used for comparison. The rate of diffusion of the membrane anchor of GRP1, which is a eukaryotic peripheral membrane protein, is 330-fold slower than the predicted rate of diffusion of the MTS [22]. The membrane anchor of GRP1 makes a specific high affinity interaction with phosphatidylinositol-3,4,5-trisphosphate (PIP_3_). Experimental work including molecular dynamics simulations suggests that the rate of diffusion of GRP1 on the membrane is limited by the frictional coefficient of PIP_3_. The much faster predicted rate of diffusion of the MTS of RNase E suggests a different mechanism of translocation. We propose that the MTS ‘glides’ in the phospholipid bilayer by making a large number of weak contacts that are rapidly broken and reformed. Although RNase E in the cell would likely have a slower rate of diffusion, our results suggest that it would have a high degree of translational freedom in the absence of interactions with other components such as membrane proteins or polyribosomes.

### Distribution and movement of RNase E

**Figure 3:**
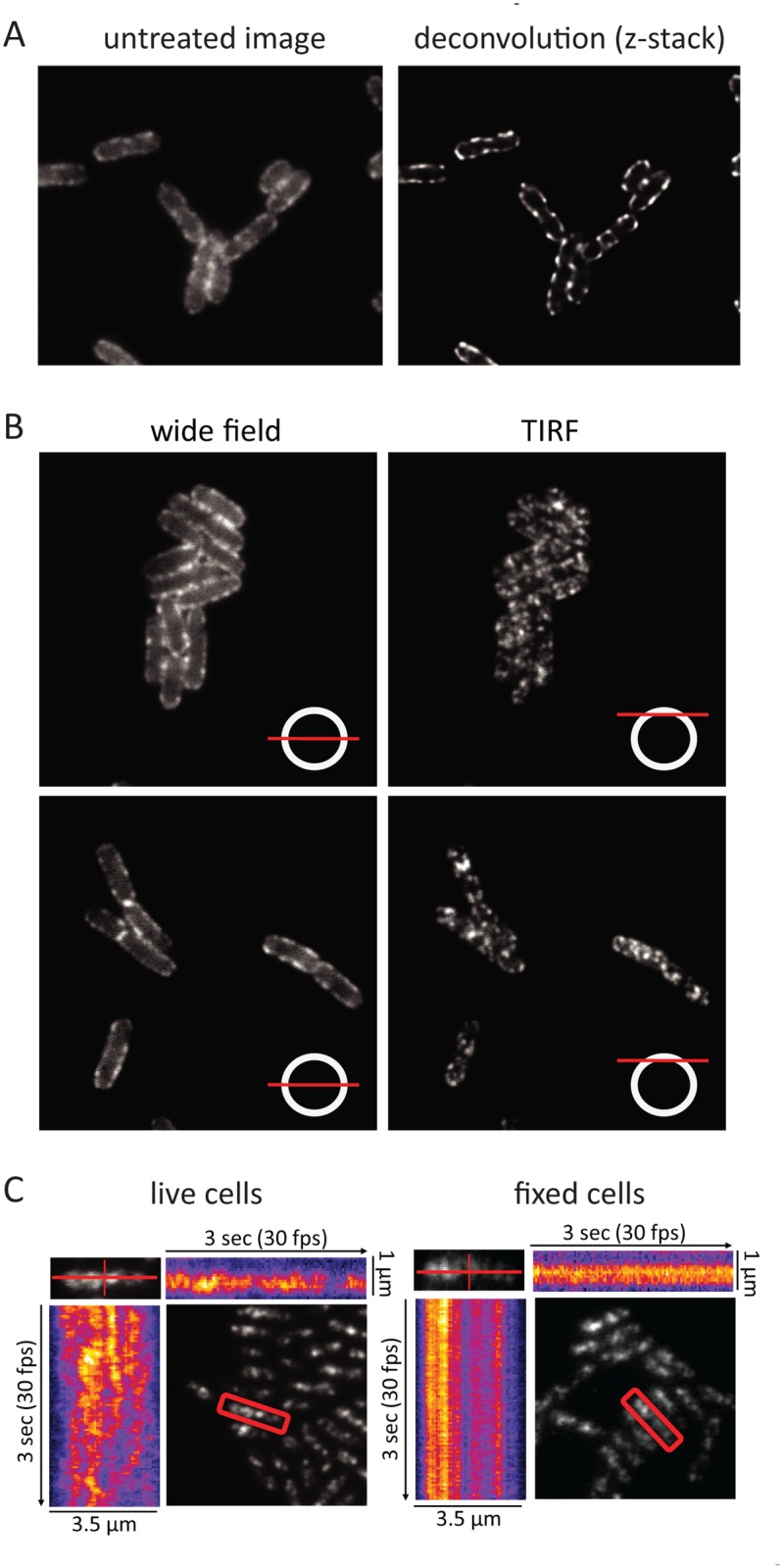
RNase E localization and mobility. Images are of the KSL2000/pVK207 strain, which expresses RNase E-YFP. Cultures were grown to mid logarithmic phase in LB at 30°C. A. Confocal microscopy of cells fixed with formaldehyde and spotted onto agarose pads. Left, image without deconvolution; right, deconvolution of the z-stack. B. Wide field and TIRF images of live cells spotted onto agarose pads. The diagram in the lower right hand corner of each image indicates the plane of focus. In these images, an exposure time of 100 ms was used to minimize distortion due to RNase E diffusion during the acquisition. C. Kymograms of RNase E mobility derived from TIRFm videos of RNase E-YFP. Left panel, live cells; right panel, formaldehyde fixed cells. The kymograms are based on a video that was made at 30 frames/sec for 3 sec (S2 Video). A single cell was scanned along its long and short axes in each frame to quantify fluorescence intensity. The scans were accumulated to generate heat maps in which the YFP signal is represented as a function of time and position in the cell.

During the cell imaging work shown in Fig. 1 and previous work with the KSL2000/pVK207 strain, which expresses RNase E-YFP (Yellow Fluorescent Protein) [6], we noticed that the RNase E fluorescence signal in live cells rapidly fluctuated, giving the impression that foci of RNase E circulate on the periphery of the cell. Fixation of the cells with formaldehyde arrests this motion. To better define the distribution of RNase E in the cell, we first examined formaldehyde fixed cells (KSL2000/pVK207) by confocal microscopy (Fig. 3A). Deconvolution of this image shows that RNase E localizes to the periphery of the cell. We then used TIRFm to selectively excite RNase E-YFP in a thin layer adjacent to the coverslip. Fig. 3B shows wide field and TIRF images of two fields of live cells. The diagram in the lower right hand corner of each image indicates the plain of focus. In these images, an exposure time of 100 ms was used to minimize motion during the acquisition, which can result in apparent but artifactual polymer-like structures. No elongated polymeric or helical structures were observed under these high-speed imaging conditions. Rather, randomly distributed clusters of RNase E were observed. S2 Fig. shows two high-speed TIRFm images of live cells taken 4 s apart. An overlay of these images artificially colored red and green shows massive redistribution of RNase E in the 4 s time interval. Using an approach that has been previously used to track MreB movement [23–25], the distribution and directionality of RNase E movement was analyzed in the kymograms shown in Fig. 3C. A single cell was scanned along its long and short axes. Heat maps derived from the intensity of the fluorescent signal were accumulated to construct the kymograms. Inspection of the fixed cell shows that the distribution of RNase E on the membrane is unchanged over a period of 3 seconds, whereas the distribution rapidly fluctuates in live cells. The lack of any clear repeat pattern in the kymograms of the live cell suggests that the movement of RNase E is random; that is, strongly correlated movement should appear as tracks or waves in the kymograms.

In the KSL2000/pVK207 strain analyzed in Fig. 3, RNase E-YFP was expressed from a low copy number plasmid that complemented a deletion of the gene encoding RNase E on the chromosome [6]. Comparable results were obtained using the Kti164 strain in which RNase E-GFP was expressed from a single copy construct on the chromosome, but the GFP signal is less intense than the YFP signal. Experimental work suggests that this difference is due to the intrinsic relative brightness of the YFP and GFP constructs since the level of RNase E-YFP and RNase E-GFP, as determined by SDS-PAGE and Western blotting is comparable to wild-type RNase E levels ([6] and S6 Fig.). We interpret the results of epifluorescence microscopy, confocal microscopy and TIRFm as evidence for the rapid diffusion of RNase E over the entire inner membrane and the formation of short-lived foci containing multiple molecules of RNase E.

### Forces acting on RNase E

**Figure 4:**
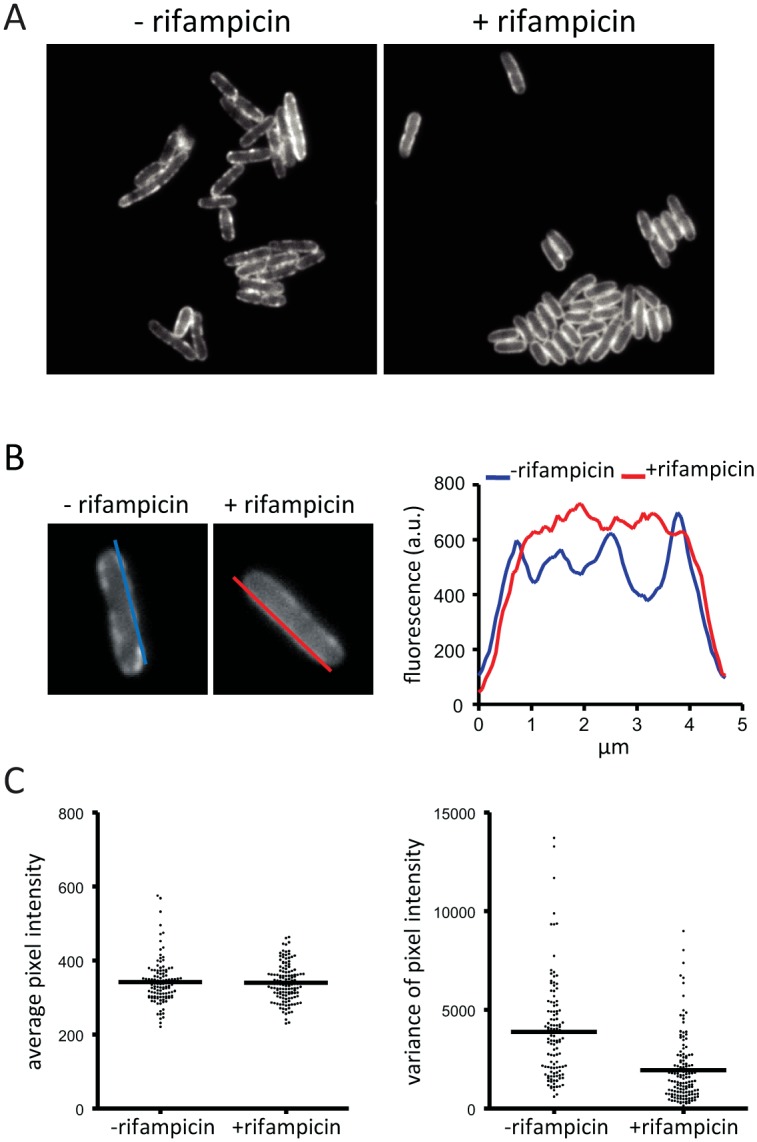
Rifampicin treatment inhibits formation of RNase E foci. Images are of the KSL2000/pVK207 strain, which expresses RNase E-YFP. Cultures were grown to mid logarithmic phase in LB at 30°C.A. Epifluorescence images showing localization of RNase E-YFP before and after treatment with rifampicin. B. Distribution of RNase E-YFP on the cytoplasmic membrane before and after treatment with rifampicin. The blue and red lines correspond to traces that were scanned to determine the fluorescence intensity. The graph shows the quantification of the scans. C. Statistical analysis of a field of cells before and after treatment with rifampicin. All cells in a field were scanned as described in panel B. Both sides of the cell were scanned except when one cell was adjacent to another. In that case, neither of the adjacent sides were scanned. A field of 83 cells (-rifampicin) yielded 118 line scans; a field of 90 cells (+rifampicin) yielded 136 line scans. The scans were analyzed to generate plots of average pixel intensity and variance in pixel intensity. The horizontal line in each plot indicates the median.

We wanted to know if the localization or diffusion of RNase E on the membrane depends on an energy source or forces generated by transcription or translation. We therefore analyzed the distribution of RNase on the membrane after treatment of the cells with carbonyl cyanide m-chlorophenyl hydrazone (CCCP), kanamycin or rifampicin. CCCP collapses the transmembrane proton gradient, while kanamycin and rifampicin are inhibitors of translation and transcription, respectively. Treatment of the cells with CCCP or kanamycin had no discernable effect on RNase E localization making it unlikely that the electrochemical gradient, ATP generation or translation influences the cellular distribution of RNase E (S7 Fig.). In contrast, after treatment with rifampicin the appearance of RNase E on the membrane is different as evidenced by the loss of foci and smooth distribution along the perimeter of the cell (Fig. 4A). The intensity of fluorescence along the membrane was measured by the line scans shown in Fig. 4B providing a method to quantify changes in the distribution of RNase E on the membrane. This analysis was applied to a field of cells to generate plots of average pixel intensity and variance (Fig. 4C). Average pixel intensity is a measure of the level of RNase E in the cell. The plot in Fig. 4C shows that the distribution of average pixel intensity is not affected by rifampicin treatment. This is expected since RNase E is a stable protein and rifampicin treatment is not predicted to change its level. In contrast, the average variance is clearly lower after treatment with rifampicin showing that the smooth distribution of RNase E along the perimeter of the cell (Fig. 4A) is a general property of a population of cells.

**Figure 5:**
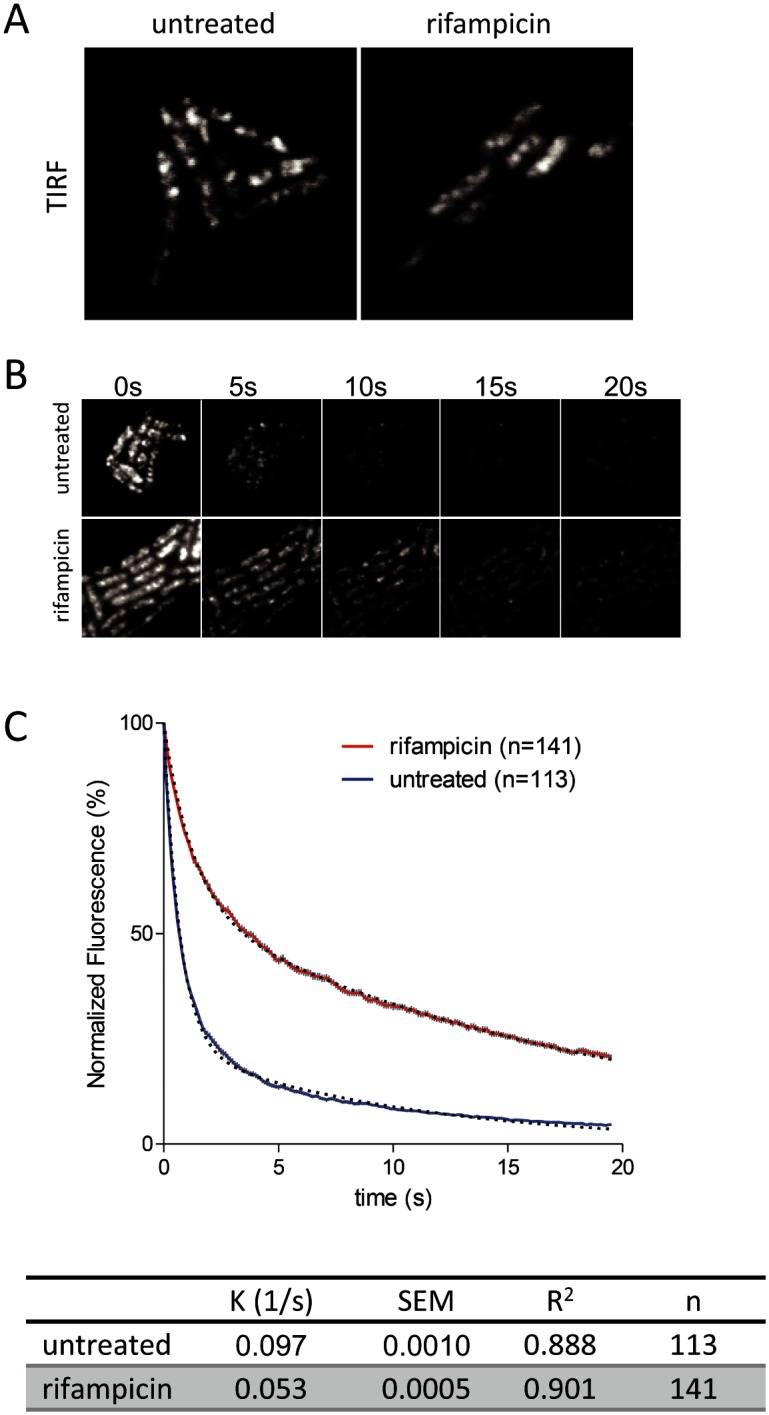
Rifampicin treatment increases the diffusion rate of RNase E. Images are of the KSL2000/pVK207 strain, which expresses RNase E-YFP. Cultures were grown to mid logarithmic phase in LB at 30°C. A. TIRFm images of live cells: 100 ms exposures, +/- 200 ug/ml rifampicin (10 min). B. TIRFm time lapse showing photobleaching: 100 ms exposures, same contrast for all images. C. Quantification of TIRFm continuous photobleaching. Data points (red or blue) and error bars (vertical gray lines) correspond to averaged, background subtracted and normalized intensities of the individual cells in the field. The number of cells in the field (n) is indicated in the upper right hand corner of the panel. Curves were fitted as two phase exponential decay using the following constraints: initial intensity = 100, decay to 0, shared fast decay rate (GFP bleach). Black dashed lines are the curve fits. The table gives the slow diffusion limited rate constant (K), standard error of the curve fit (SEM), goodness of fit (R^2^) and number of cells analyzed (n). See [21] for more detail regarding determination of relative diffusion rates.

To further examine the effect of rifampicin, we used the intrinsic photobleaching that occurs in TIRFm to measure the diffusion of RNase E on the membrane. Briefly, since only a portion of the membrane is excited in TIRFm, the rate of photobleaching is related to the rate of diffusion of the fluorescent protein. If a fluorescent protein diffuses rapidly relative to the intrinsic rate of photobleaching, then the pool of bleachable molecules will be slowly depleted since individual molecules only spend a short time on the surface that is excited. If, on the other hand, diffusion is slow, then the pool of bleachable molecules will be depleted faster since individual molecules will spend a longer time on the surface that is excited. With the appropriate controls, it is possible to estimate a relative rate of diffusion by this technique [21]. Fig. 5A shows snapshots of RNase E distribution by TIRFm before and after treatment with rifampicin, Fig. 5B is a photobleaching time course and Fig. 5C is the quantification of the photobleaching experiment. Rifampicin treatment results in a diffuse distribution of RNase E with few if any intense foci as compared to the untreated control. Remarkably, rifampicin decreases the rate of photobleaching, indicating an increase in the rate of RNase E diffusion. To test if rifampicin treatment has a general effect on the diffusion of membrane proteins, we measured the photobleaching rate of the F1Fo ATP synthase before and after treatment with rifampicin (S8 Fig.). As the rate of diffusion of the F1Fo ATP synthase is not affected, we conclude that rifampicin specifically affects RNase E diffusion.

### Requirement of RNA substrate to form RNase E foci

**Figure 6:**
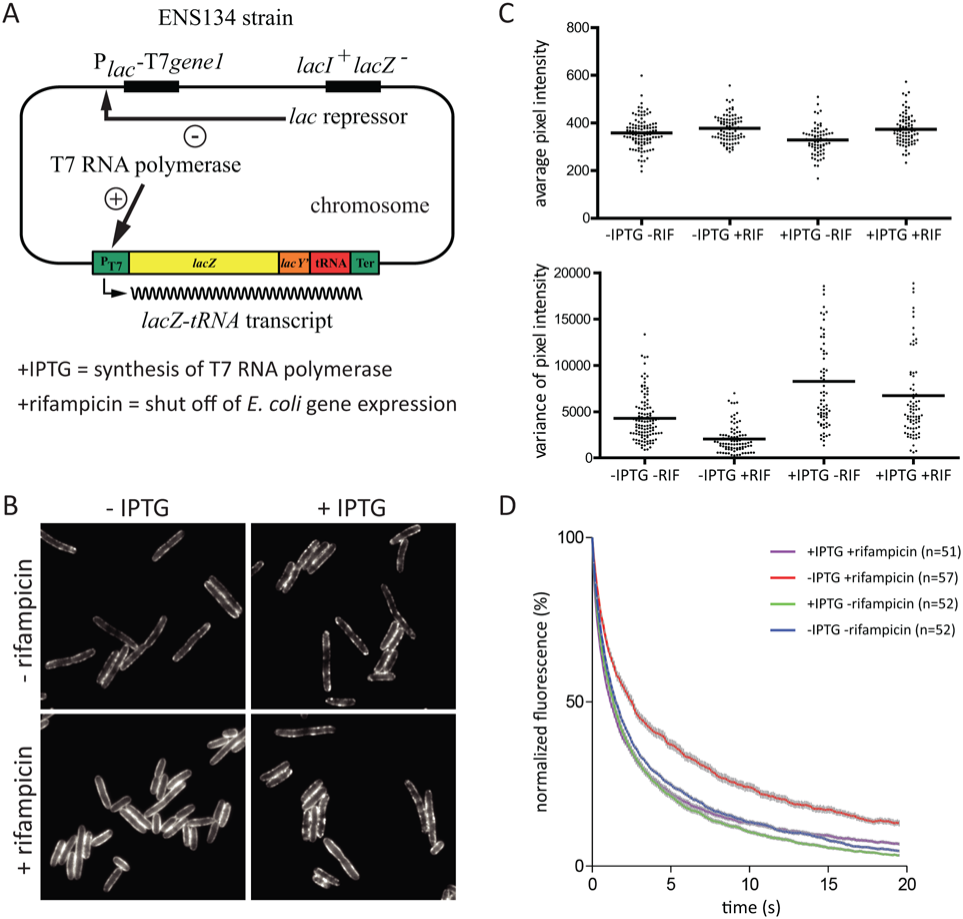
The effect rifampicin on the distribution and diffusion of RNase E is due to the absence of RNA substrate. Images are of the ENS134/pVK207 strain, which expresses RNase E-YFP. Cultures were grown to mid logarithmic phase in LB at 30°C. A. Relevant features of the ENS134 strain. The expression of the bacteriophage T7 RNA polymerase, which is under the control of the *lac* repressor, can be induced with IPTG. The normal chromosomal copy of the *lacZ* gene is inactivated. The *lacZ-tRNA* transcript is under the control of a bacteriophage T7 promoter. The induction of T7 RNA polymerase synthesis by IPTG leads to transcription of *lacZ-tRNA*. If rifampicin is added after induction of T7 RNA polymerase, then *E. coli* transcription is shut off, but *lacZ-tRNA* synthesis continues since the T7 RNA polymerase is insensitive to rifampicin. RNase E acts on the *lacZ-tRNA* in two processes: it initiates the degradation of the *lacZ* mRNA and it is involved in the maturation of the tRNA. B. Epifluorescence images of cells after treatment with IPTG (1 mM, 10 min) and/or rifampicin (200 µg/ml, 10 min). In experiments where both compounds were added, the cells were treated first with IPTG, then with rifampicin. C. Analysis of cell fields corresponding to the conditions in panel B. The plots showing average pixel intensity and the variance were generated as described in Fig. 4. The horizontal lines indicate the median of average pixel intensity or variance. D. Quantification of TIRFm continuous photobleaching as described in Fig. 5. In the upper right hand corner, the different conditions are color coded; n indicates the number of cells that were in the field.

To explore whether RNA substrate is required to form RNase E foci, we exploited the ENS134 strain encoding bacteriophage T7 RNA polymerase [26, 27], which is insensitive to inhibition by rifampicin. In this strain, it is possible to inhibit *E. coli* RNA polymerase under conditions in which T7 RNA polymerase actively transcribes genes with a bacteriophage T7 promoter. The ENS134 strain has a chromosomal copy of the gene for T7 RNA polymerase under the control of an inducible *lac* promoter, and a chromosomal copy of the gene encoding *lacZ* under the control of a bacteriophage T7 promoter (Fig. 6A). The *lacZ* gene is fused to a tRNA gene followed by a transcription terminator. Transcription by T7 RNA polymerase results in high level synthesis of a *lacZ-tRNA* transcript. RNase E is necessary for degradation of the *lacZ* mRNA [27]. Maturation of the tRNA is predicted to require RNase E and RNase P considering the established pathway in *E. coli* [28]. In order to visualize RNase E in the ENS134 strain, we introduced pVK207, which is the low copy number plasmid encoding the RNase E-YFP fusion used in the work shown in Figs. 3, 4 and 5. Fig. 6B shows epifluorescence images of the ENS134/pVK207 strain. The control panel (-rifampicin, -IPTG) shows that RNase E-YFP distribution is comparable to what we observed in the KLS2000/pVK207 strain. Autoregulation of RNase E expression, which is predicted to down regulate expression of plasmid *rne-yfp* and chromosomal *rne* genes, results in a level of total RNase E (RNase E + RNase E-YFP) comparable to the normal RNase E level [6, 29, 30]. Since there are approximately 5 plasmid copies of *rne-yfp* for each chromosomal copy of *rne*, RNase E-YFP is the predominant form of RNase E in these cells.

Images in Fig. 6B show that cells treated first with IPTG and then rifampicin have a pattern of peripheral RNase E foci similar to cells that have not been treated with rifampicin (+IPTG, +rifampicin vs. +IPTG, ‑rifampicin). This result suggests that synthesis of the *lacZ-tRNA* transcript in the presence of rifampicin results in an interaction that stimulates the formation of RNase E foci. The effect of rifampicin on a field of cells was quantified (Fig. 6C). This result shows that changes in distribution of RNase E along the perimeter of the cell is a general property of the population of cells treated with rifampicin. The small reduction in variance after treatment with rifampicin (+IPTG, +rifampicin vs. +IPTG, ‑rifampicin) is likely due to inhibition of *E. coli* RNA polymerase. From these results we conclude that the effect of rifampicin on formation of RNase E foci is not a secondary consequence of the shut off of *E. coli* transcription. We next analyzed RNase E-YFP photobleaching. Fig. 6D shows that there is no effect on the rate of diffusion of RNase E if IPTG is added before rifampicin. This result suggests that synthesis of the *lacZ-tRNA* transcript in the presence of rifampicin results in an interaction that constrains the diffusion of RNase E. Taken together, these results show that there is a direct correlation between formation of foci and constraints on the diffusion of RNase E. Considering this experimental work, we propose that the formation of RNase E foci requires interaction with RNA substrate, that foci formation constrains the diffusion of RNase E, and that rifampicin acts on the foci indirectly by depleting the pool of RNA substrate.

## Discussion

Live cell microscopy shows that RNase E is located on the cytoplasmic membrane and that the DEAD-box RNA helicase RhlB is associated with RNase E, but neither protein forms cytoskeletal-like structures as reported earlier [12, 13]. This is the first report in which RhlB has been visualized directly in live cells as previous work employed indirect immunofluorescence. The YFP, GFP and CFP tags used here are A206K variants that have been reported to minimize dimerization [31]. The fusion proteins are present at levels comparable to wild type. We have used agarose pads that do not disturb the physiology of the cell, whereas cells immobilized on glass slides were imaged in the work showing cytoskeletal-like structures. Under the conditions used here, RNase E is highly mobile over the entire surface of the membrane. The movement is spontaneous since it does not appear to be driven by an energy dependent process suggesting that the motion of degradosome assembly is due to continuous buffeting by other macromolecules in the densely packed milieu of the cell including the membrane-associated cell wall synthesis machinery [21]. Recent biophysical studies of live bacterial cells suggest that particle size has a disproportionate influence on diffusion. Small particles including free ribosomes and multienzyme complexes can be treated as components in a liquid-like state whereas larger particles such as polyribosomes are apparently constrained in a solid-like state that requires metabolic activity for ‘mixing’ [32, 33].

Molecular dynamics simulations provide a conceptual framework for visualizing how the MTS anchors RNase E to the inner cytoplasmic membrane by a novel mechanism permitting a high degree of translational freedom. Although the interaction of an individual MTS with the membrane represents a weak force compared to integral membrane proteins with multiple trans-membrane segments, the cumulative effect of having four MTS elements spatially co-localized as a consequence of RNase E tetramerization should have a strong binding effect through chelate cooperativity [34], which is consistent with biochemical work showing that the solubilization and purification of RNase E requires high concentrations of nonionic detergent [35]. Considering previous measurements of the interaction of a peptide corresponding to the MTS with a phospholipid bilayer composed of *E. coli* lipids (K_d_ = 1.3 × 10^-6^ M) [6] and the effect of chelate cooperativity, we believe that RNase E should be fully membrane-bound, which is consistent with the confocal microscope image presented here. Membrane association could aid the organization of the RNase E tetramer and bring the noncatalytic C-terminal region, which interacts with the other components of the RNA degradosome, within a restricted hemisphere in which they may cooperate with the catalytic core of RNase E. The interaction of the MTS with the membrane is predicted to affect the local concentration of lipid species. The spatial co-localization of the four MTS elements in the RNase E tetramer could accentuate a preference for anionic lipids at the site of membrane docking. Cooperativity in the interaction between anionic lipids with basic residues flanking the hydrophobic core of four MTS elements could further increase avidity of RNase E for the membrane.

We have described a highly dynamic distribution on the membrane in which RNase E forms short-lived foci. Analysis of the dynamics of RNase E motion by TIRFm suggests that diffusion on the membrane is random with no indication of correlation with the long or short axis of the cell. This result excludes models in which RNase E moves along ‘tracks’ in the membrane or is constrained by the machinery involved in cell wall synthesis as has recently been described for MreB [23, 24]. Depletion of RNA substrates by inhibition of transcription results in disappearance of foci and an increase in the rate of diffusion of RNase E. Our results suggest that foci are sites of degradation in which several molecules of RNase E as well as other components of the RNA degradosome interact with an RNA substrate. RNase E foci could therefore have a function analogous to eukaryotic P-bodies and stress granules, which are ribonucleoprotein particles containing factors involved in translation inhibition and mRNA degradation [36, 37]. RNase E foci are nevertheless much smaller and much more short-lived than P-bodies or stress granules.

An important activity of RNase E is general and regulated mRNA degradation. An example of regulated mRNA degradation is the rapid response associated with quorum sensing in *Vibrio cholera*, which involves sRNA, Hfq and RNase E [38]. A caveat to such regulation is that the target mRNA finds its way to RNase E and is actively degraded. Recent work suggests that RNase E can interact with polyribosomes and sRNA/Hfq complexes [39, 40]. It can therefore be envisaged that RNase E associated with an sRNA/Hfq complex could interrogate a polyribosome as mRNA spools through the translational machinery providing a window of opportunity for degradation if the sRNA can match a recognition region in the transcript. General mRNA degradation, which is initiated in the absence of a regulatory factor, could also involve polyribosome interrogation with direct competition between translation re-initiation and cleavage by RNase E. In either case, a productive interaction would lead to the formation of ribosome-free mRNA facilitating the recruitment of additional RNase E molecules. Sequestration of the RNA degradosome to the two dimensional surface of the inner cytoplasmic membrane and rapid diffusion could increase cooperativity in general and regulated mRNA degradation as well as other processes involving RNase E such as tRNA maturation. It is also possible that this system has a quality control function in the degradation of defective transcripts that fail to form polyribosomes or other ribonucleoprotein complexes. Characterization of RNA substrates localized to RNase E clusters could help to identify cellular processes that are facilitated by the formation of cooperative degradation bodies.

## Materials and Methods

### Atomistic and coarse grain molecular dynamics simulations

Atomistic simulations were performed using the GROMACS simulation package, version 4.5.1 [41] with the GROMOS53a6 force-field [42, 43] and the SPC water model [44]. Simulations were performed in the NPT ensemble, with the Nosé Hoover thermostat [45, 46] with a time constant of 0.5 ps and the Parrinello—Rahman barostat [47] with a time constant of 5.0 ps used to maintain a temperature of 310 K and a pressure of 1 bar. Long-range electrostatic interactions were treated using the smooth particle mesh Ewald method and a long-range dispersion correction was applied to the energy and pressure beyond the cut-off. The neighbor list was updated every 5 steps during the simulations. All bonds were constrained using the LINCS algorithm [48] allowing a 2 fs timestep to be applied in all simulations. The protonation states of all titratable residues of the peptides were assigned using pH 7. Repeats of all of the simulations were performed using different randomly assigned starting velocities. Peptides were manually positioned in the bulk solvent just above the lipid headgroups of one leaflet of the pre-equilibrated DPPC lipid bilayers.

Within the GROMACS simulation package, the MARTINI force-field was used for the lipids and a variant of this was used to represent the interactions between lipid and protein [49]. The peptide secondary structure was retained using weak restraints between backbone particles to represent hydrogen-bonds, as has been shown to work well for other peptides [50]. The different bilayers (*E. coli* membrane models contained 14 cardiolipin, 88 DPPE, and 26 DPPG lipid molecules) were setup by substituting the appropriate lipids into pre-equilibrated DPPC bilayers. The non-bonded neighbor list was updated every 10 steps. The integration time step was 40 ps. All simulations were performed at constant temperature (310 K) and pressure (1 bar), using the Berendsen thermostat and barostat [51]. Lennard-Jones interactions were shifted to zero between 9 Å and 12 Å, and electrostatics were shifted to zero between 0 Å to 12 Å, with a relative dielectric constant of 15.

### Strains construction

Strains and plasmids are listed in Table 1. The Kti series of strains was constructed in the NCM3416 background using the λ Red recombination system [52, 53]. PCR products (S1 Table) were transformed into NCM3416/pKD20. Cm^r^ (Chloramphenicol resistant) transformants were selected at 30°C and then streaked at 42°C to eliminate pKD20. The constructs were purified by bacteriophage P1 transduction of the Cm^r^ marker into NMC3416. The strains were then transformed with pCP20 and ampicillin resistant colonies selected at 30°C. To eliminate pCD20 and the Cm^r^ cassette, transformants were streaked at 43°C on ampicillin and then tested for loss of the Cm^r^ marker. The coding sequence of the fusion protein and flanking regions were then sequenced. All constructs are C-terminal fusions and the FRT scar is located downstream of the translation stop codon. For construction of the strains expressing RNase E-mCherry, RNase E-GFP and RhlB-CFP, DNA fragments generated by crossover PCR were transformed into NCM3416/pKD20. For construction of strains expressing RNase E(∆MTS)-mCherry, RNase E(∆Sca)-mCherry and RNase E(∆HBS)-mCherry, plasmids containing the mutant *rne* allele fused to mCherry and the Cm^r^ cassette were constructed (S1 Table). These plasmids were then used as templates to produce PCR products to transform NCM3416/pKD20.

**Table 1:** Strains and plasmids.

	**Characteristics**	**Reference**
**Strains**		
KSL2000/pVK207	*rne-yfp*	[6]
NCM3416	*E. coli* K12, F^-^, λ^-^, *zib-207*::Tn*10*	[16]
PBRN1	*rne(Y25am)*, amber mutation	[60]
ENS134	*E. coli* BL21(DE3), *lacZ::Tn10*, *malP*p*∆534::P_T7_lacZ-Arg5*	[26]
EB2	*atpB-gfp*	G. Deckers-Hebestreit
Kti162	NCM3416, *rne-mch* ^a^	this work
Kti164	NCM3416, *rne-gfp*	this work
Kti491	MCM3416, *rne(∆mts)-gfp*	this work
Kti194	NCM3416, *rhlB-cfp*	this work
Kti200	NCM3416, *rne-mch*, *rhlB-cfp*	this work
Kti211	NCM3416, *rne-mch*, *rhlB-cfp*, *∆pcnB*	this work
Kti230	NCM3416, *rne-mch*, *∆rhlB*	this work
Kti240	NCM3416, *rne-mch*, *∆rhlB*, *pcnB^-^*	this work
Kti513	NCM3416, *rne(∆mts)-mch*	this work
Kti515	NCM3416, *rne(∆mts)-mch*, *rhlB-cfp*	this work
Kti663	NCM3416, *∆rhlB*	this work
Kti682	NCM3416, *rne(∆hbs)-mch*	this work
Kti688	NCM3416, *rne(∆sca)-mch*	this work
Kti738	NCM3416, *rne(∆hbs)-mch*, *rhlB-cpf*	this work
Kti740	NCM3416, *rne(∆sca)-mch*, *rhlB-cfp*	this work
**Plasmids**		
pRSet-mCherry	*mCherry*	[61]
pKD20	ts replication, ampicillin resistance, λ Red recombinase	[52]
pCP20	ts replication, ampicillin resistance, FLP recombinase	[52]
pSAB11	pSC101*ori*, spectinomycin resistance	[15]
pDAG739	*gfp-frt-cat-frt*	[62]
pLP62^b^	pSAB11-*rne(Y25am)*	this work
pJMK5	pLP62-*rne(∆sca)-mch-frt-cat-frt*	this work
pJMK6	pLP62-*rne(∆hbs)-mch-frt-cat-frt*	this work
pJMK7	pLP62-*rhlB-cfp-frt-cat-frt*	this work
pKti10	pLP62-*rne(∆mts)-mch-frt-cat-frt*	this work

^a^ *mch* = *mCherry*.

^b^ The *rne* alleles of the JMK and Kti derivatives of this plasmid are wild type at codon 25 (Y25).

Deletions in RNase E variants are as follows (wild type RNase E coordinates): ∆MTS, 567–582; ∆scaffold, 702–1061; ∆HBS, 705–737. SDS-PAGE showed that the fusion proteins are stable and their levels are comparable to the wild type protein. For Western blotting, the transfer of RNase E-GFP and RNase E-mCherry is inefficient and not sufficiently reproducible to be quantified. We therefore visualized the fusion proteins directly by SDS-PAGE and Coomassie staining (S6A–S6B Fig.), which is possible because the fusions are amongst the largest proteins in the cell and they migrate as distinct bands. As it is difficult to raise specific high-titer antibodies against RhlB, we examined RhlB and RhlB-CFP using affinity purified polyclonal rabbit antibody as described [54] or antibody against CFP (S6C–S6D Fig.). These results show that RhlB-CFP is stable and that its level in the cell is comparable to wild type RhlB. Strains expressing defective RNase E variants grow slower than an isogenic wild type control and the defective variant is expressed at a higher level due to autoregulation [6, 29, 30]. Since the growth rate and RNase E levels of the strains expressing RNase E-mCherry, RNase E-GFP and RNase E-YFP are normal (S6 Fig. and [6]), we conclude that these fusion proteins are fully active. RhlB is not essential, but its activity can be tested *in vivo* since *∆rhlB* in a *pcnB-* background results in the accumulation of mRNA degradation intermediates [55]. RhlB is part of a 3′ exonucleolytic mRNA degradation pathway involving RhlB and PNPase as components of the RNA degradosome. S9 Fig. shows a Northern blot probed for an mRNA degradation intermediate known to accumulate in the *∆rhlB*, *pcnB-* background. A probe for 5S ribosomal RNA was included as a loading control. In the *pcnB-* background, the mRNA degradation intermediate accumulates in the *rhlB-cfp* strain, but the level is much higher in the *∆rhlB* strain. We therefore conclude that the RhlB-CFP fusion is active *in vivo* albeit at a lower level than wild type RhlB.

### Cell growth and microscopy

Liquid cultures were grown at 30°C with vigorous aeration in LB or MOPS medium [56] supplemented with glycerol (0.5%) and amino acids (50 µg/ml each l-amino acid except tryptophane, tyrosine and phenylalanine). Microscope setups are listed in S2 Table. Bacteria were prepared for microscopy as described [57]. Aliquots (0.5–1.0 µl) from cultures grown to mid logarithmic phase were spotted on microscope slides covered with a thin layer of agarose (1.2% in water). After a few minutes to allow absorption of the cells, the agarose pad was covered with a slip and the slide immediately mounted on the microscope to take images. Preparation of the microscope slide and imaging was performed at room temperature. To chemically fix cells, aliquots from cultures grown to mid logarithmic phase were treated with formaldehyde (1%) for 10 min at 30°C with agitation and then quenched with glycine (100 mM). Images were analyzed using ImageJ [58, 59]. TIRFm photobleaching and quantitative analyses to determine relative diffusion rates were performed as described [21].

For the analysis of peptide binding, FITC-labelled PAQPGLLSRFFGALKALFSGGK (wild type) or PAQPGLLSR***AA***GALKALFSGGK (F574A/F574A variant) were mixed with liposomes and visualized by spinning disk confocal fluorescence microscopy. Liposomes were prepared from *E. coli* lipid extracts (Avanti Polar Lipids) using Octyl-Glucoside detergent dialysis method as described [57]. Liposomes were diluted to 1 mg/ml in 5 mM Tris pH 7.5, 150 mM KCl, and peptides were added at concentration of 100 ug/ml. 2 mg/ml BSA was included as unspecific protein. Incubation (5 min) and microscopy were performed at 30°C.

### Ethics statement

Not applicable. This study did not involve human participants, specimens or tissue samples, or vertebrate animals, embryos or tissues.

## Supporting Information

S1 Fig Membrane localization of RhlB depends on a direct protein-to-protein interaction with RNase E. Wide field images of cells expressing RNase E-mCherry and RhlB-CFP. PC = phase contrast. See Fig. 1 for further details.
(TIF)

S2 Fig High speed TIRFm images of KSL2000/pVK207 cells (RNase E-YFP). Exposures (100 ms) were taken 4 s apart. The images were artificially colored green and red to create the merged image.
(TIF)

S3 Fig Lipid-protein contacts with the wild type (WT) MTS and variants (AA, E and P) predicted from molecular dynamics simulations. CARD, cardiolipin; PE, dipalmitoylphosphatidylethanolamine; PG, dipalmitoylphosphatidylglycerol.
(TIF)

S4 Fig Energy of interaction of the wild type MTS and variants (AA, E and P) with the phospholipid bilayer predicted from molecular dynamics simulations. (TIF)

S5 Fig Preferential interaction of the MTS with anionic lipids predicted from molecular dynamics simulations. Backbone of peptide, cyan; cardiolipin, orange; PE (dipalmitoylphosphatidylethanolamine), blue; PG (dipalmitoylphosphatidylglycerol), yellow.
(TIF)

S6 Fig SDS-PAGE and Western blotting analyses of fusion protein stability and levels. From the results in this figure, we conclude that the RNase E-GFP, RNase E-mCherry and RhlB-CFP fusion proteins are stable and that their level in the cell is comparable to their wild type counterpart. A. Whole-cell extracts from strains expressing wild type RNase E (*rne^+^*), RNase E-GFP (*rne-gfp*) or RNase E(∆MTS)-GFP (*rne(∆mts)-gfp*) were separated by SDS-PAGE and visualized by Coomassie brilliant blue staining. The positions of RNase E (NMC3416) and RNase E-GFP (Kti164) are indicated by arrows. The fusion proteins migrate slower than their actual molecular weight due to atypical amino acid composition of the scaffold region of RNase E. Although RNase E(∆MTS)-GFP contains a small deletion, it reproducibly migrates slightly slower than its RNase E-GFP counterpart. Positions of size markers (Kd) are shown to right. B. Whole cell extracts containing RNase E variants fused to mCherry were separated as in panel A. Wild type RNase E (NCM3416) and RNase E-mCherry (Kti162) are indicated by arrows. The RNase E(∆Sca)-mCherry variant (Kti688) does not migrate as a distinct band in this gel. Western blotting showed that the RNase E(∆Sca)-mCherry variant migrates with an apparent molecular weight of approximately 120 kDa. C. Whole cell extracts from *∆rhlB*, *rhlB^+^* or *rhlB-cf*p strains were separated by SDS-PAGE then electroblotted to a Hybond-C Extra filter, which was probed with an affinity-purified rabbit polyclonal antibody raised against RhlB. The position of RhlB and RhlB-CFP are indicated by arrows; a non-specific signal present in all lanes is indicated by an asterisk. RhlB is difficult to detect, which is likely due to difficulties in raising specific high-titer antibodies against bacterial DEAD-box RNA helicases. Nevertheless, the *∆rhlB* strain permits identification of wild type RhlB and RhlB-CFP. D. Whole cell extracts were separated and electroblotted as in panel C. The filter was probed with antibody against GFP, which cross-reacts with CFP. The arrow indicates the position of the RhlB-CFP fusion protein. pAM-*rhlB-cfp* is a low copy number plasmid containing the gene for the RhlB-CFP fusion protein under endogenous expression signals. The transformation of pAM-*rhlB-cfp* into Kti200, Kti515, Kti738 and Kti740 results in higher levels of RhlB-CFP. Proteolysis resulting in free CFP (∼30 kDa) is not detected.
(TIF)

S7 Fig RNase E localization after treatment of cells with CCCP and kanamycin. Images are of the KSL2000/pVK207 strain, which expresses RNase E-YFP. PC = phase contrast. Growth conditions and microscopy are as described in Fig. 4. The cells were treated with CCCP (100 µm) for 2 min or kanamycin (100 µg/ml) for 10 min.
(TIF)

S8 Fig TIRFm photobleaching of RNase E-YFP and AtpB-GFP. AtpB is a subunit of the F1Fo ATP synthase. Treatment with rifampicin (200 µg/ml) was for 10 min. The curves were generated from a field of cell as described in Fig. 5. These results show that the rate of diffusion of the F1Fo ATP synthase is not affected by rifampicin. As the rate of diffusion of the F1Fo ATP synthase is not affected, we conclude that rifampicin specifically affects RNase E diffusion.
(TIF)

S9 Fig Test of RhlB-CFP activity in vivo. Northern blot showing the accumulation of an mRNA degradation product (REP) in the *∆rhlB*, *pcnB-* background as described previously [53]. The mRNA degradation intermediates is derived from a REP (Repeated Extragenic Palindrome) element located in the *sucB-sucC* transcription unit. The blot was also probed for 5S ribosomal RNA as a loading control. The REP degradation intermediate accumulates in the *rhlB-cfp* strain, but the level is much higher in the *∆rhlB* strain. We therefore conclude that the RhlB-CFP fusion is active *in vivo* albeit at a lower level than wild type RhlB.
(TIF)

S1 Table Primers, templates and PCR products used in strain construction. (DOCX)

S2 Table Microscope setups used for epifluorescence and TIRF microscopy. (DOCX)

S1 Video Movie showing coarse grain molecular dynamics simulation of MTS peptide interaction with phospholipid bilayer. (MPG)

S2 Video TIRFm video of RNase E-YFP. Live cells, left; cells fixed with formaldehyde, right. This video, which is played at half speed, was used to make the kymograms in Fig. 3B.
(AVI)
