# Supplementary figures and images for: Membrane Recognition and Dynamics of the RNA Degradosome
Source: PLoS Genet. 2015 Feb 3;11(2):e1004961. doi: 10.1371/journal.pgen.1004961 (PMC4372235; doi:10.1371/journal.pgen.1004961)

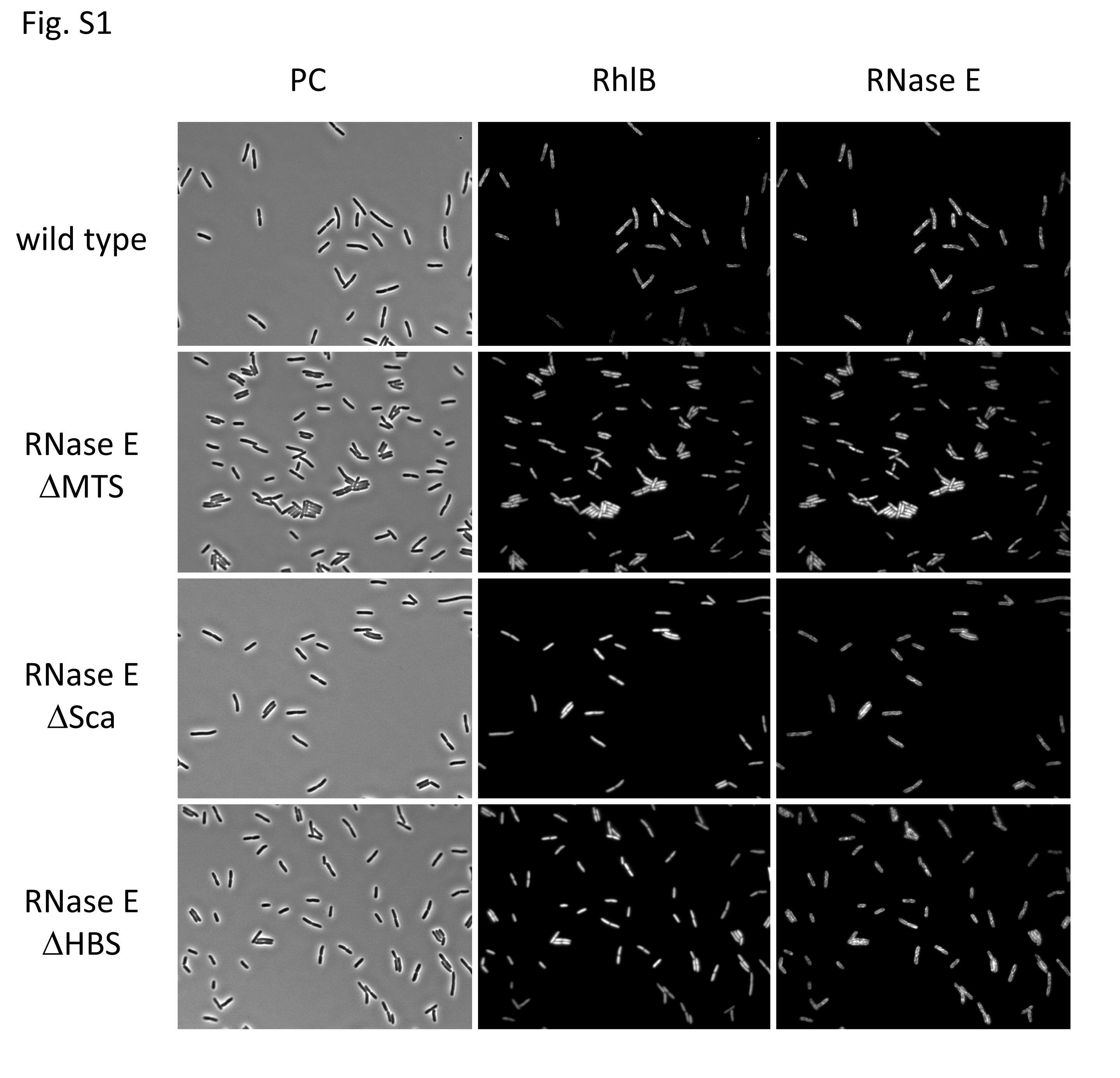

Supplement: S1 Fig — Wide field images of cells expressing RNase E-mCherry and RhlB-CFP. PC = phase contrast. See Fig. 1 for further details. (TIF) [file pgen.1004961.s001.tif]

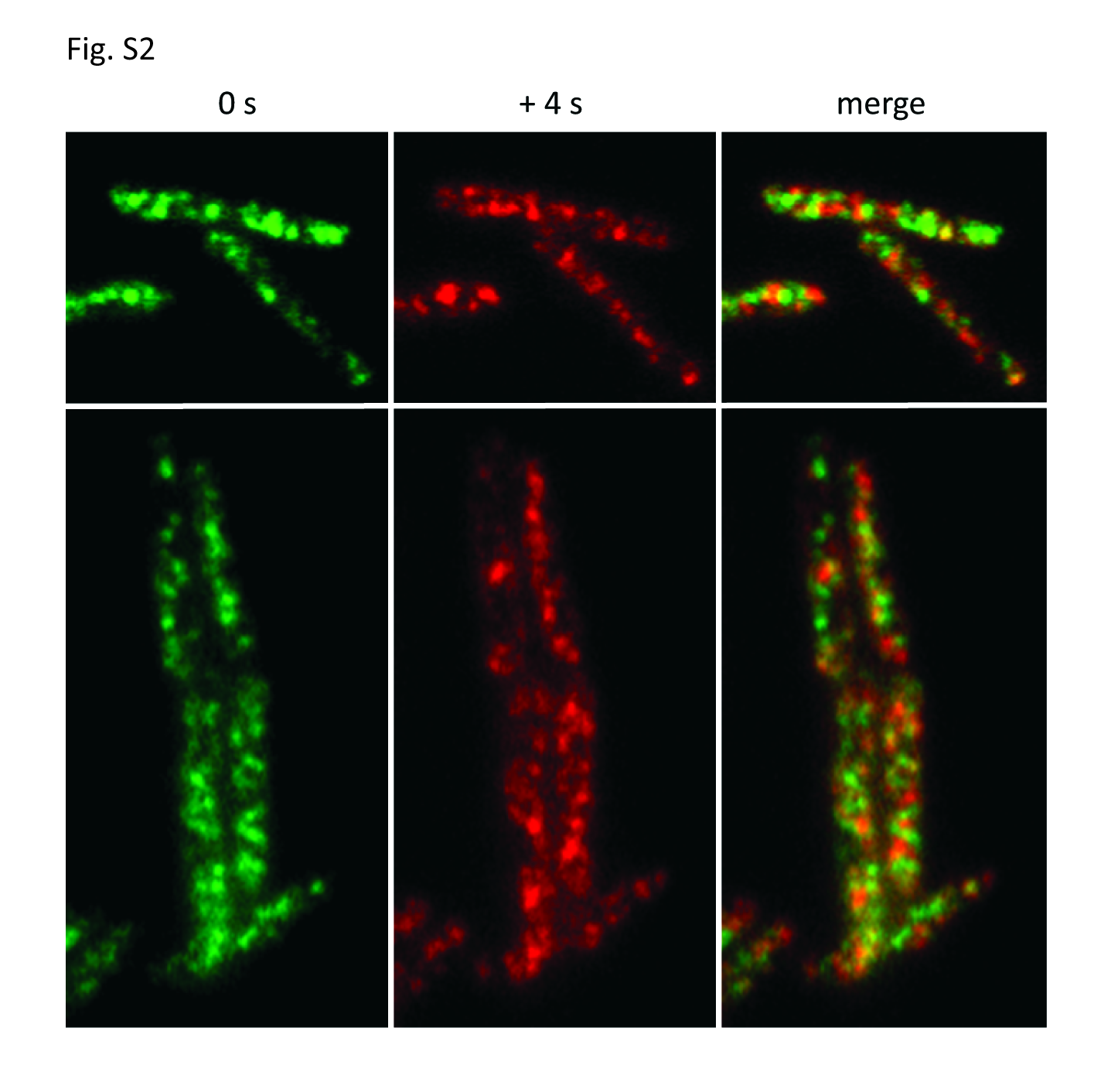

Supplement: S2 Fig — Exposures (100 ms) were taken 4 s apart. The images were artificially colored green and red to create the merged image. (TIF) [file pgen.1004961.s002.tif]

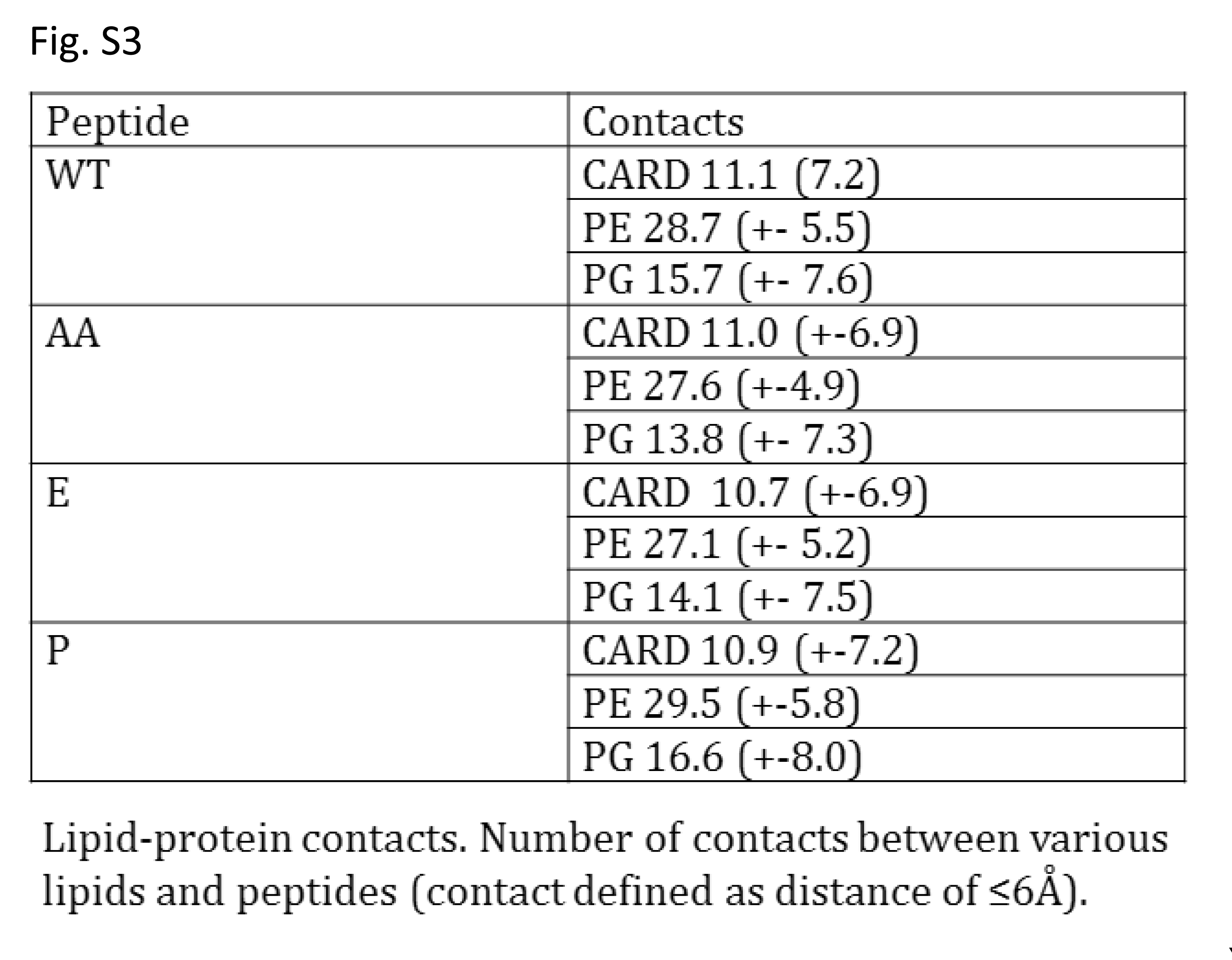

Supplement: S3 Fig — CARD, cardiolipin; PE, dipalmitoylphosphatidylethanolamine; PG, dipalmitoylphosphatidylglycerol. (TIF) [file pgen.1004961.s003.tif]

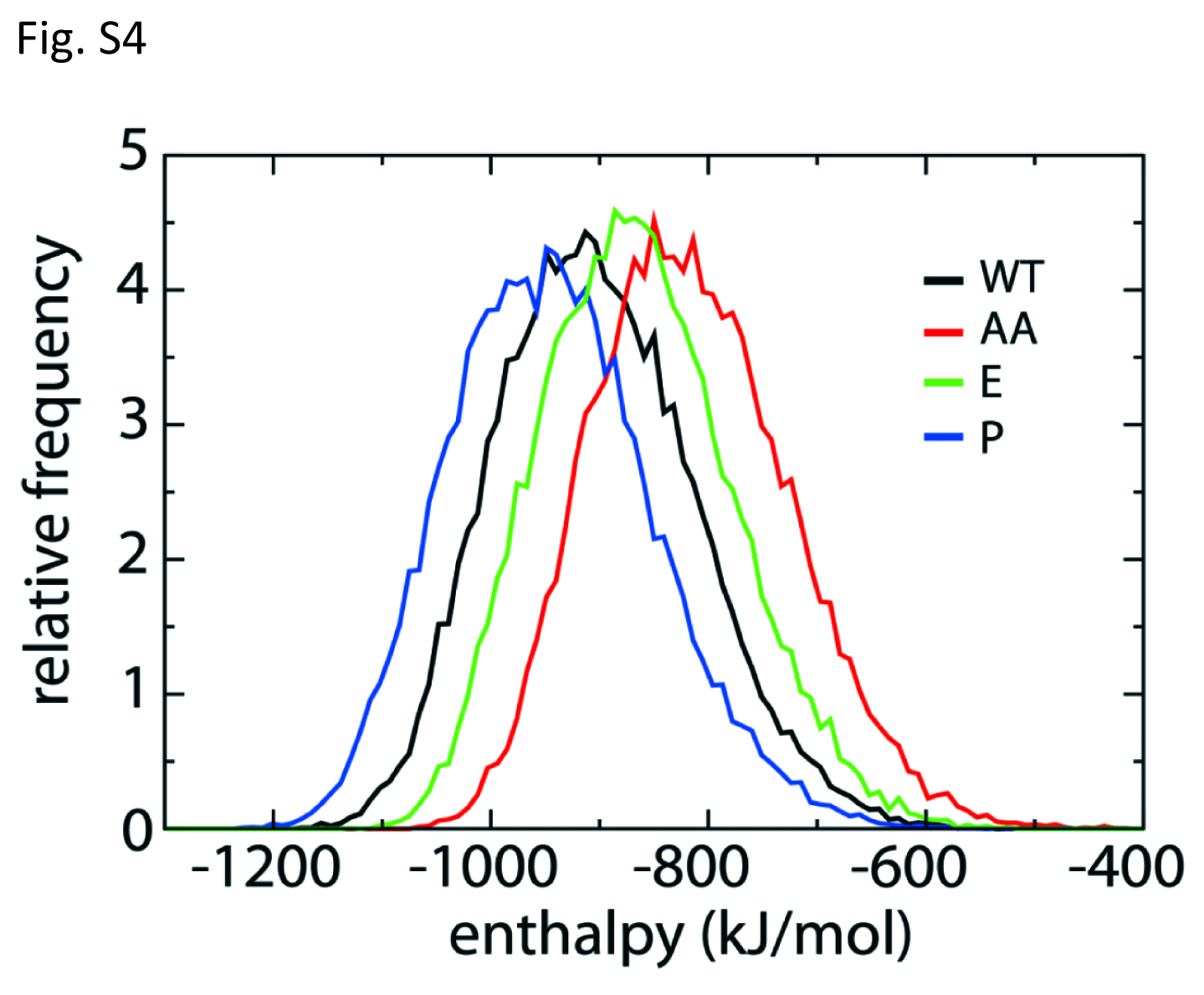

Supplement: S4 Fig — (TIF) [file pgen.1004961.s004.tif]

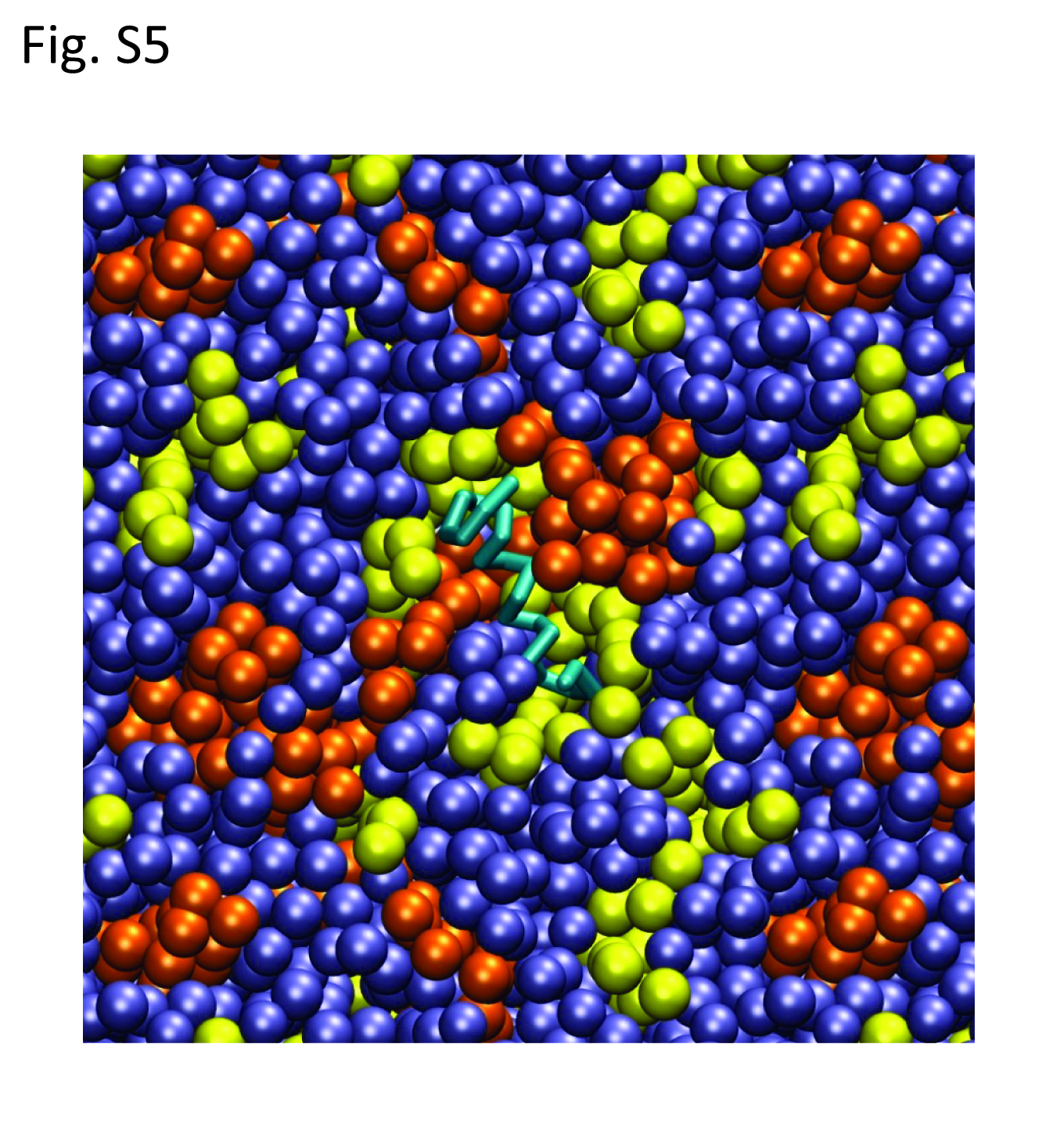

Supplement: S5 Fig — Backbone of peptide, cyan; cardiolipin, orange; PE (dipalmitoylphosphatidylethanolamine), blue; PG (dipalmitoylphosphatidylglycerol), yellow. (TIF) [file pgen.1004961.s005.tif]

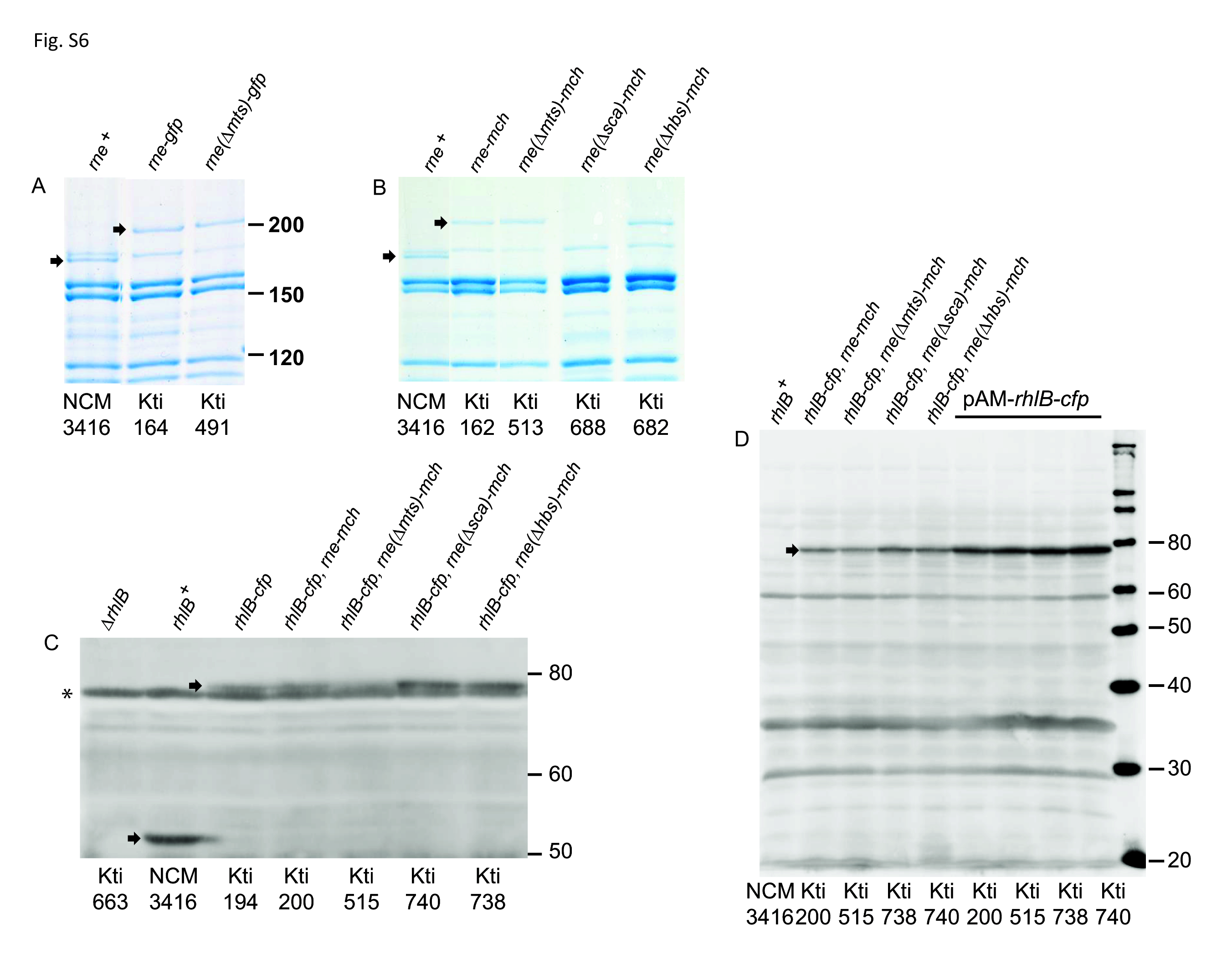

Supplement: S6 Fig — From the results in this figure, we conclude that the RNase E-GFP, RNase E-mCherry and RhlB-CFP fusion proteins are stable and that their level in the cell is comparable to their wild type counterpart. A. Whole-cell extracts from strains expressing wild type RNase E (rne+), RNase E-GFP (rne-gfp) or RNase E(∆MTS)-GFP (rne(∆mts)-gfp) were separated by SDS-PAGE and visualized by Coomassie brilliant blue staining. The positions of RNase E (NMC3416) and RNase E-GFP (Kti164) are indicated by arrows. The fusion proteins migrate slower than their actual molecular weight due to atypical amino acid composition of the scaffold region of RNase E. Although RNase E(∆MTS)-GFP contains a small deletion, it reproducibly migrates slightly slower than its RNase E-GFP counterpart. Positions of size markers (Kd) are shown to right. B. Whole cell extracts containing RNase E variants fused to mCherry were separated as in panel A. Wild type RNase E (NCM3416) and RNase E-mCherry (Kti162) are indicated by arrows. The RNase E(∆Sca)-mCherry variant (Kti688) does not migrate as a distinct band in this gel. Western blotting showed that the RNase E(∆Sca)-mCherry variant migrates with an apparent molecular weight of approximately 120 kDa. C. Whole cell extracts from ∆rhlB, rhlB+ or rhlB-cfp strains were separated by SDS-PAGE then electroblotted to a Hybond-C Extra filter, which was probed with an affinity-purified rabbit polyclonal antibody raised against RhlB. The position of RhlB and RhlB-CFP are indicated by arrows; a non-specific signal present in all lanes is indicated by an asterisk. RhlB is difficult to detect, which is likely due to difficulties in raising specific high-titer antibodies against bacterial DEAD-box RNA helicases. Nevertheless, the ∆rhlB strain permits identification of wild type RhlB and RhlB-CFP. D. Whole cell extracts were separated and electroblotted as in panel C. The filter was probed with antibody against GFP, which cross-reacts with CFP. The arrow indicates the positi [file pgen.1004961.s006.tif]

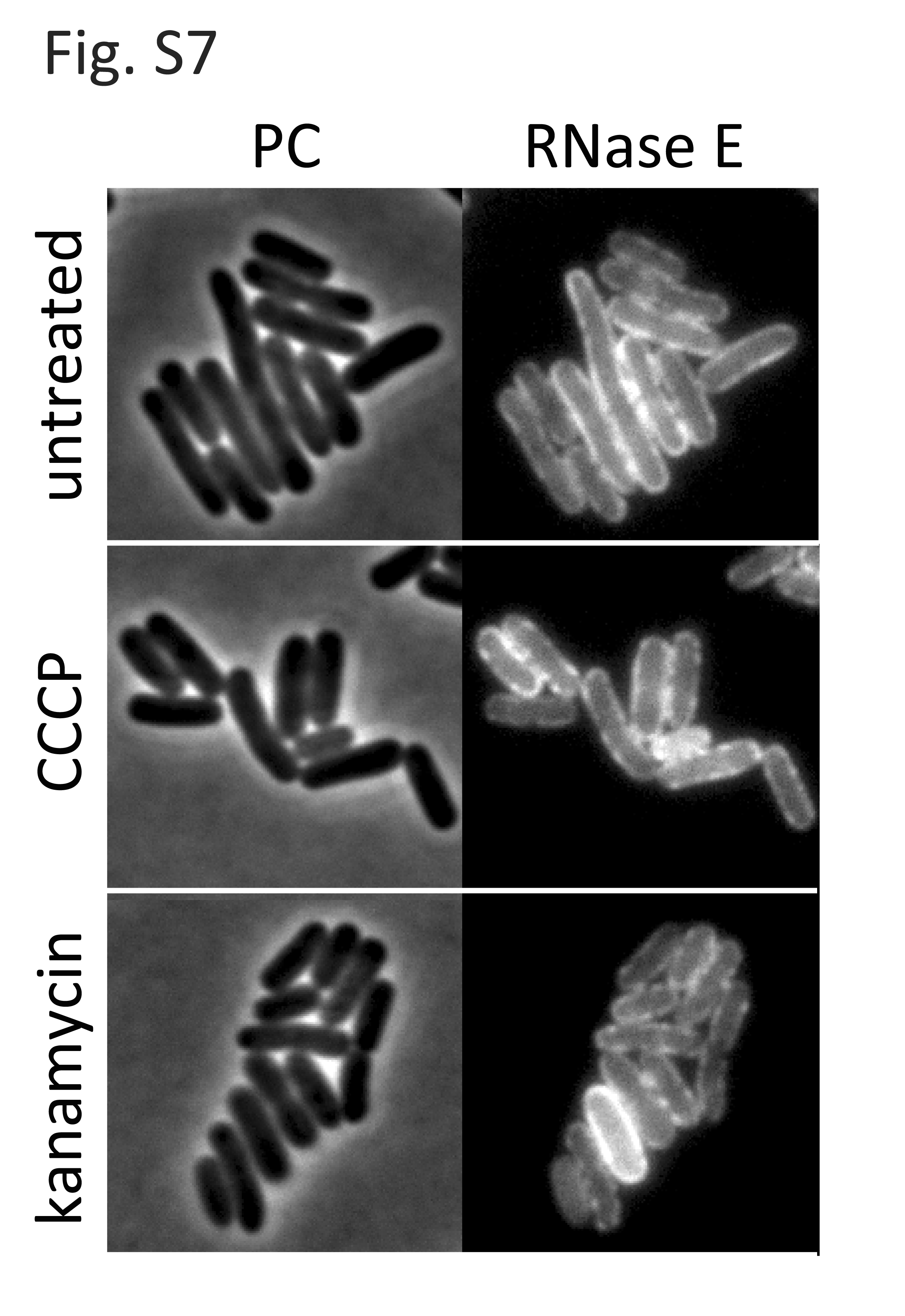

Supplement: S7 Fig — Images are of the KSL2000/pVK207 strain, which expresses RNase E-YFP. PC = phase contrast. Growth conditions and microscopy are as described in Fig. 4. The cells were treated with CCCP (100 µm) for 2 min or kanamycin (100 µg/ml) for 10 min. (TIF) [file pgen.1004961.s007.tif]

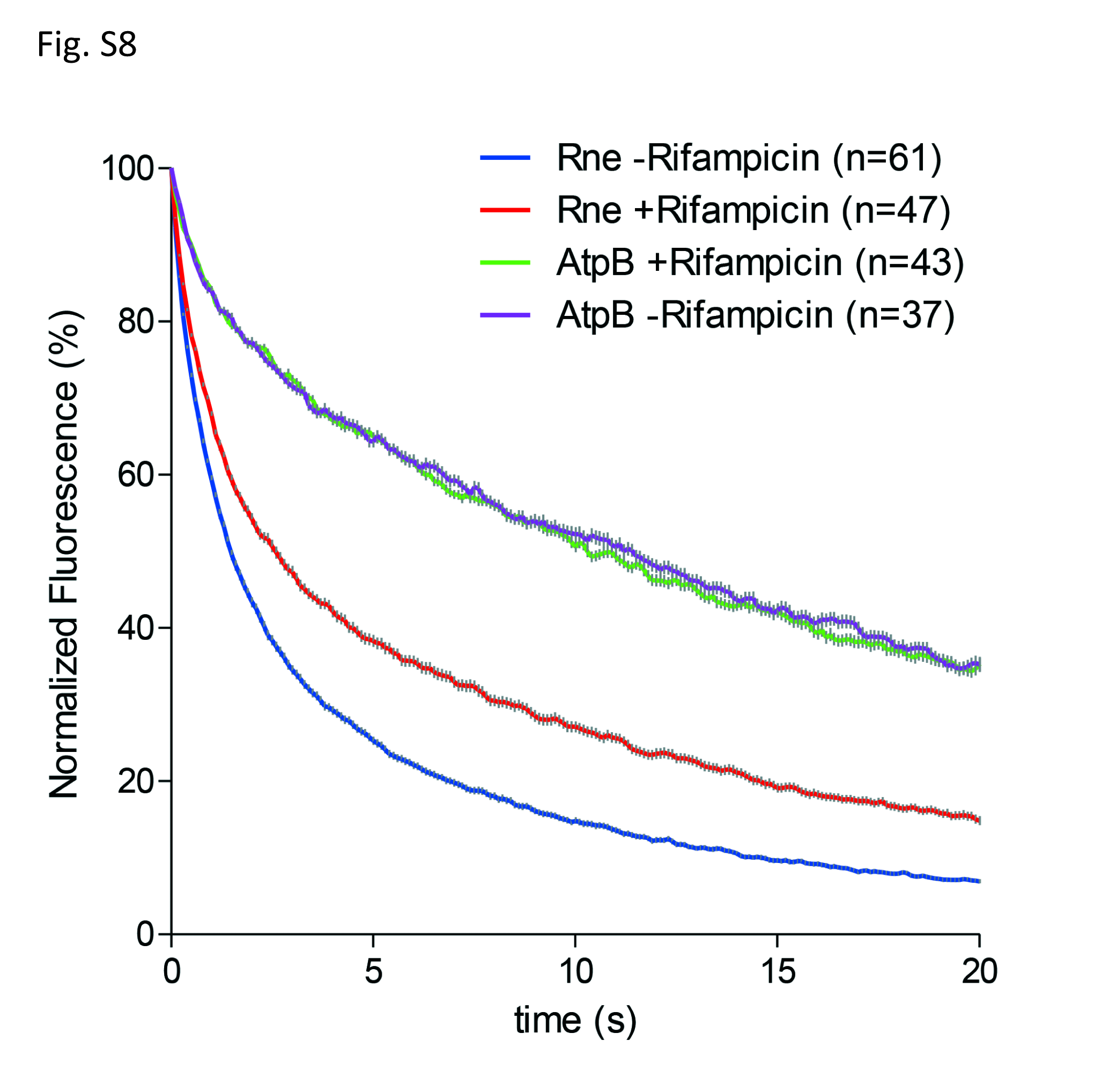

Supplement: S8 Fig — AtpB is a subunit of the F1Fo ATP synthase. Treatment with rifampicin (200 µg/ml) was for 10 min. The curves were generated from a field of cell as described in Fig. 5. These results show that the rate of diffusion of the F1Fo ATP synthase is not affected by rifampicin. As the rate of diffusion of the F1Fo ATP synthase is not affected, we conclude that rifampicin specifically affects RNase E diffusion. (TIF) [file pgen.1004961.s008.tif]

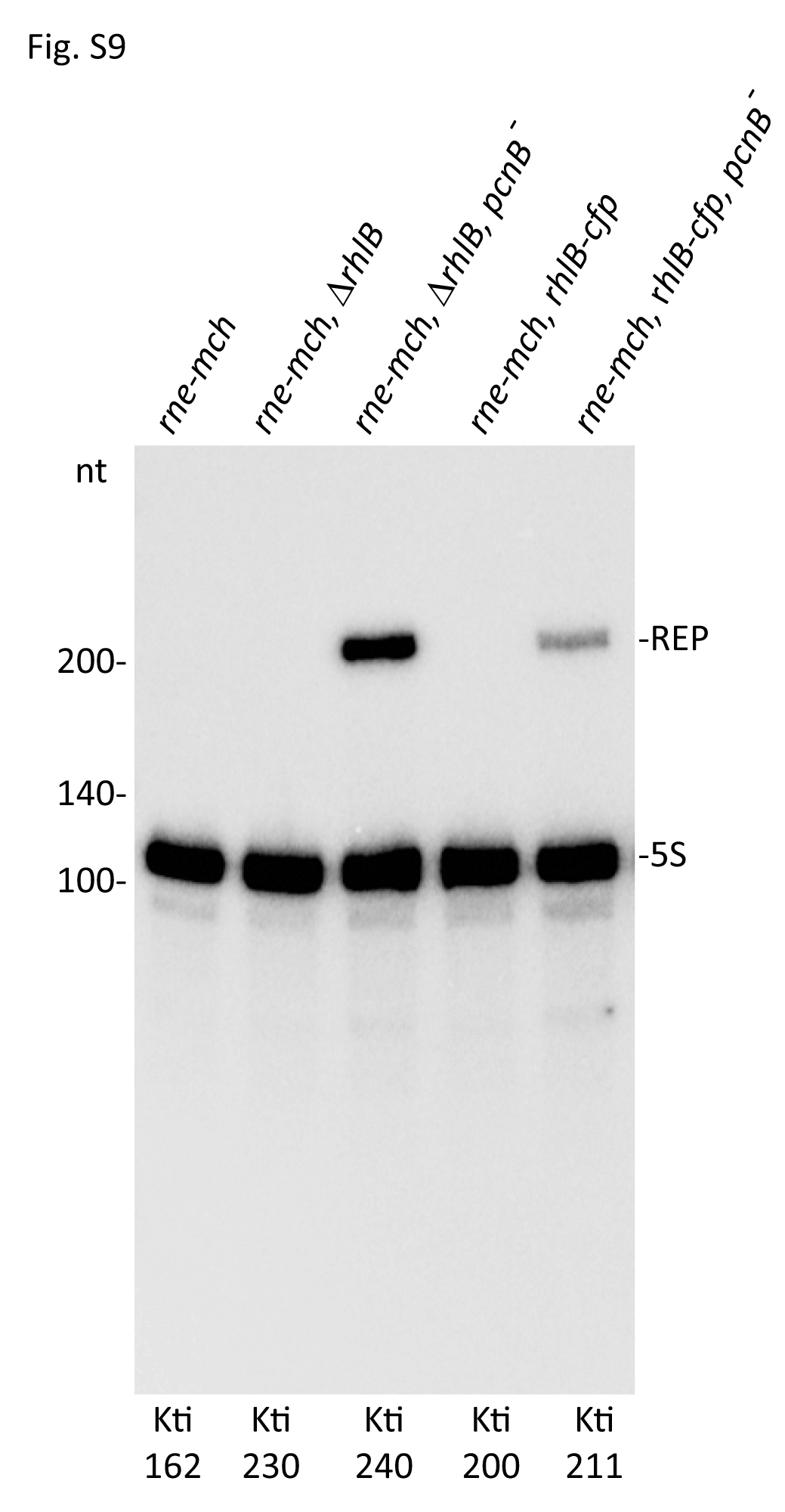

Supplement: S9 Fig — Northern blot showing the accumulation of an mRNA degradation product (REP) in the ∆rhlB, pcnB- background as described previously [53]. The mRNA degradation intermediates is derived from a REP (Repeated Extragenic Palindrome) element located in the sucB-sucC transcription unit. The blot was also probed for 5S ribosomal RNA as a loading control. The REP degradation intermediate accumulates in the rhlB-cfp strain, but the level is much higher in the ∆rhlB strain. We therefore conclude that the RhlB-CFP fusion is active in vivo albeit at a lower level than wild type RhlB. (TIF) [file pgen.1004961.s009.tif]
