# Supplementary material for: Membrane Recognition and Dynamics of the RNA Degradosome
Source: PLoS Genet. 2015 Feb 3;11(2):e1004961. doi: 10.1371/journal.pgen.1004961 (PMC4372235; doi:10.1371/journal.pgen.1004961)
Supplement: S1 Table — (DOCX) [file pgen.1004961.s010.docx]

**Table S1.** Primers and PCR products.

| **Primer** | **Sequence (5’-3’)** |
| --- | --- |
| OKT1 | tcatgcctctgccgctcctgcgcgtccgcaacctgttgagatggtgagcaagggcgagga |
| OKT2 | aagcagctccagcctacacaatcgctcaagttacttgtacagctcgtcca |
| OKT3 | accggcggcatggacgagctgtacaagtaacttgagcgattgtgtaggct |
| OKT4 | ccctggcagttaccagggcttgattactttgagctaattatccatccagtctattatgaa |
| OKT7 | tcatgcctctgccgctcctgcgcgtccgcaacctgttgagatggtgagcaagggcgagga |
| OKT14 | cccgcgcacaggcaatggtccgcgtcgtactggcgctccg |
| OKT17 | ttgatacagtttgaatgattttgagtatgacattttttattccatccagtctattatgaa |
| OKT18 | tcaggcgcaacaagaagcgaaggcgctgaatgttgaagagatggtgagcaagggcgagga |
| OKT19 | gtaccgccgacgagcagcaagcgccgcgtcgtgaacgtagccgccgccgtaatgatgata |
| MTS up | ttgtagctccagcaccgaaagctgcaccggcaacaccagcagctcctgca |
| **Primer pair** | **Template/PCR product** |
| OKT1/OKT2 | pRSet-mCherry/*mCherry* |
| OKT3/OKT4 | pDAG739/*frt-cat-frt* |
| OKT1/OKT4 | *mCherry* + *frt-cat-frt*/*mCherry-frt-cat-frt* |
| OKT4/OKT7 | pDAG739/*gfp-frt-cat-frt* |
| OKT14/OKT17 | pJMK7/*cfp-frt-cat-frt* |
| MTS up/OKT4 | pKti10/*rne(∆mts)-mCherry-frt-cat-frt* |
| OTK19/OKT4 | pJMK6/*rne(∆hbs)-mCherry-frt-cat-frt* |
| OKT18/OKT4 | pJMK5/*rne(∆sca)-mCherry-frt-cat-frt* |
