# Supplementary material for: Membrane Recognition and Dynamics of the RNA Degradosome
Source: PLoS Genet. 2015 Feb 3;11(2):e1004961. doi: 10.1371/journal.pgen.1004961 (PMC4372235; doi:10.1371/journal.pgen.1004961)
Supplement: S2 Table — (DOCX) [file pgen.1004961.s011.docx]

**Table S2.** Microscope setups.

| Microscope | Objective | Filters | Camera | Light source | Software | Add-ons |
| --- | --- | --- | --- | --- | --- | --- |
| Nikon Ti-E/B with perfect focus system (PFS) | Nikon Plan Apo DM 100x PH-3 ON1.4 | GFP (Ex: 482BP35; DM: 506; Em: 536BP40)  YFP (Ex: 500BP24; DM: 520; Em: 542BP27)  CFP (Ex: 438BP24; DM: 458; Em: 483BP32),  mCherry (Ex : 562BP40; DM: 593; Em : 641BP75)  (Semrock) | OrcaR2 digital CCD camera (Hamamatsu) | Nikon Intensilight 130W High-Pressure Mercury Lamp | Nis-Elements AR | - |
| Zeiss  Axiovert 200M | Zeiss Plan- Neofluar 100x PH3 NA 1.3 | CFP (49001; Ex 430/24x; Dm 455; Em 470/24m)  YFP (49003; Ex 500/20x; Dm 515; Em 535/30x)  mCherry (49008; Ex 560/40x; Dm 585; Em 630/75x)  (Chroma) | Coolsnap HQ2 (Photometrics) | Lambda LS (Sutter Instruments) | Metamorph 6 (Molecular Devices, Inc) | - |
| Nikon Eclipse Ti Spinning Disk Confocal | Nikon Apo VC 100x NA1.40 | ZT440-445/488- 491/594 rpc (Chroma) | Rolera EM-C2 (QImaging) | Calypso 491 nm DPSS laser (Cobolt) | Frap-AI 7.7.5.0 (MAG Biosystems) | CSU22 spinning disk unit (Yokogawa) |
| Nikon N-SIM/N-STORM | Nikon CFI APO TIRF 100x NA 1.49 | TRF49904 (Chroma) | IXON X3 (Andor) | Argon Ion multiline laser (Melles Griot) | NIS-Elements 4.0 | TIRF illumination module (Nikon) |
